# Significance of oxygen transport through aquaporins

**DOI:** 10.1038/srep40411

**Published:** 2017-01-12

**Authors:** Janusz J. Zwiazek, Hao Xu, Xiangfeng Tan, Alfonso Navarro-Ródenas, Asunción Morte

**Affiliations:** 1 Department of Renewable Resources, University of Alberta, Edmonton, AB, T6G 2E3, Canada; 2 Departamento de Biología Vegetal (Botánica), Facultad de Biología, Universidad de Murcia, Campus de Espinardo, 30100 Murcia, Spain

## Abstract

Aquaporins are membrane integral proteins responsible for the transmembrane transport of water and other small neutral molecules. Despite their well-acknowledged importance in water transport, their significance in gas transport processes remains unclear. Growing evidence points to the involvement of plant aquaporins in CO_2_ delivery for photosynthesis. The role of these channel proteins in the transport of O_2_ and other gases may also be more important than previously envisioned. In this study, we examined O_2_ permeability of various human, plant, and fungal aquaporins by co-expressing heterologous aquaporin and myoglobin in yeast. Two of the most promising O_2_-transporters (*Homo sapiens* AQP1 and *Nicotiana tabacum* PIP1;3) were confirmed to facilitate O_2_ transport in the spectrophotometric assay using yeast protoplasts. The over-expression of NtPIP1;3 in yeasts significantly increased their O_2_ uptake rates in suspension culture. In *N. tabacum* roots subjected to hypoxic hydroponic conditions, the transcript levels of the O_2_-transporting aquaporin NtPIP1;3 significantly increased after the seven-day hypoxia treatment, which was accompanied by the increase of ATP levels in the apical root segments. Our results suggest that the functional significance of aquaporin-mediated O_2_ transport and the possibility of controlling the rate of transmembrane O_2_ transport should be further explored.

Since the discoveries that membrane intrinsic proteins (MIPs) are involved in transmembrane water transport^1^,^2^, evidence has been growing that links different members of the aquaporin family to transport processes of other small neutral molecules, including CO_2_^3^,^4^,^5^. The transport of these molecules has been associated with fundamental physiological processes^3^,^6^. Similarly to the long-prevailing views of water transport, a possible significance of pore-mediated transport for CO_2_ and O_2_ has been sometimes downplayed due to theoretical and experimental evidence suggesting rapid diffusion of these gases through the lipid bilayer^7^,^8^. While functional significance of aquaporin-mediated CO_2_ transport has been demonstrated for photosynthesis and cell signaling processes^3^,^6^, the importance of pore-mediated O_2_ transport to transcellular O_2_ fluxes and cell function remains elusive^9^,^10^,^11^.

In the present study, we used the yeast cell system (*Saccharomyces cerevisiae* INVSc1, Invitrogen) to co-express sperm whale (*Physeter macrocephalus*) myoglobin^12^ in the yeast expression vector pAG425GAL-ccdB together with one of the 20 different aquaporins from human, plants, or fungi (Supplementary Information Notes S1 and S2) in the vector pAG426GAL-ccdB, to evaluate the impact of heterologous aquaporin expression on myoglobin oxygenation as an indicator for O_2_ permeability of the yeast plasma membrane. We also examined the transcript abundance of plasma membrane intrinsic proteins (PIPs) in relation to ATP levels in the roots of *Nicotiana tabacum* under hypoxia in hydroponic culture in order to evaluate possible functional significance of the O_2_-transporting aquaporins.

## Results

### Protein expression and transcript abundance of myoglobin and aquaporins

**Figure 1:**
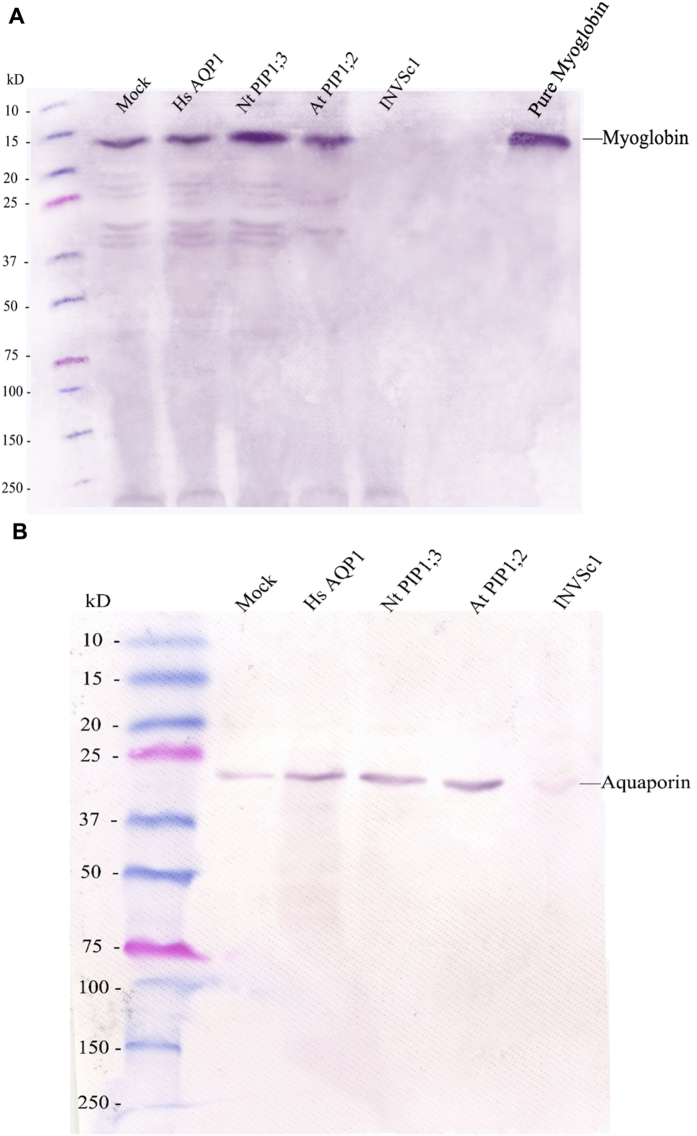
Immunoblot probed with the anti-myoglobin antibody and the anti-human aquaporin 1 antibody. (**A**) The yeast total proteins were immunoblotted with the primary anti-myoglobin antibody. (**B**) The yeast total proteins were immunoblotted with the primary anti-human aquaporin 1 antibody.

Immunoblotting with anti-myoglobin antibody demonstrated the presence of myoglobin in the selected yeast strains that were constructed to express myoglobin, but not in INVSc1 (Fig. 1A). Quantitative RT-PCR showed that transcript abundance of myoglobin was similar in the transformed yeast strains (Fig. S1). Immunoblotting with anti-human aquaporin 1 antibody demonstrated that the antibody recognized the expressed heterologous aquaporins *Homo sapiens* HsAQP1, *Nicotiana tabacum* NtPIP1;3, and *Arabidopsis thaliana* AtPIP1;2 in the respective strains, and also, weakly, the yeast homologous aquaporins in the mock strain constructed to express myoglobin only (Fig. 1B). The qRT-PCR assay with higher specificity than immunoblotting showed that the transcript abundance of the heterologously-expressed aquaporin genes *HsAQP1, NtPIP1*;*3* and *AtPIP1*;*2* was negligible in the mock strain, but significantly high in each corresponding strain (Fig. S1).

**Figure 2:**
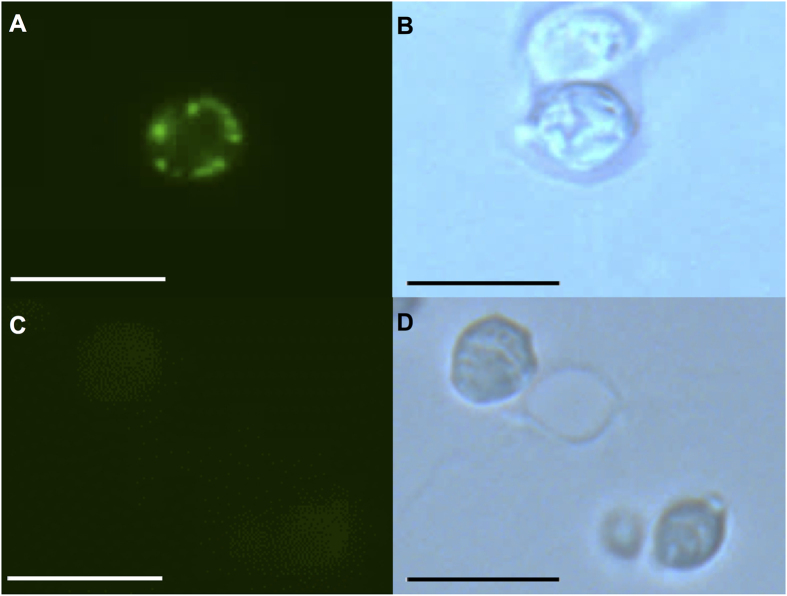
Indirect immunofluorescence of paraffin-embedded yeast cells of HsAQP1 strain after the incubation with the primary anti-aquaporin 1 monoclonal antibody and the fluorescein-conjugated secondary antibody. (**A**) HsAQP1 strain under blue light excitation. (**B**) HsAQP1 strain in bright field. (**C**) Mock strain under blue light excitation. (**D**) Mock strain in bright field. The length of bars is 10 μm.

Following formaldehyde fixation^13^, paraffin embedding and preparation of sectioned yeast cells for immunodetection with the anti-human aquaporin 1 antibody, strong immunofluorescence was detected in the periphery of the yeast cell section of the HsAQP1 strain in comparison with the relatively weak intracellular fluorescence signal (Fig. 2), pointing to the plasma membrane as the likely localization site. This is consistent with the subcellular localization prediction by TargetP^14^, suggesting the absence of mitochondrial targeting peptide and pointing to the secretory pathway as the most likely location of HsAQP1 in eukaryotic cells (Supplementary Information Table S1).

### O_2_ transport

Of the yeast strains that were examined, those expressing HsAQP1, NtPIP1;3 NtPIP1;4, NtPIP2;1, and NtXIP1;1 showed statistically significant increases in O_2_ permeability with preliminary spectrophotometric measurements as evidenced by higher rates of change in myoglobin A_541_ absorbance (Figs S2 and S3A) compared with mock control (Fig. S3B). Over-expression of the *A. thaliana* (AtPIP1;1, AtPIP1;2, AtPIP 1;3, AtPIP1;4, and AtPIP2;1) and *Laccaria bicolor* (LbAQP1, LbAQP3, LbAQP5, LbAQP6, and LbAQP7) aquaporins did not alter A_541_ absorbance (Fig. S3B), indicating no significant effect on O_2_ permeability.

**Figure 3:**
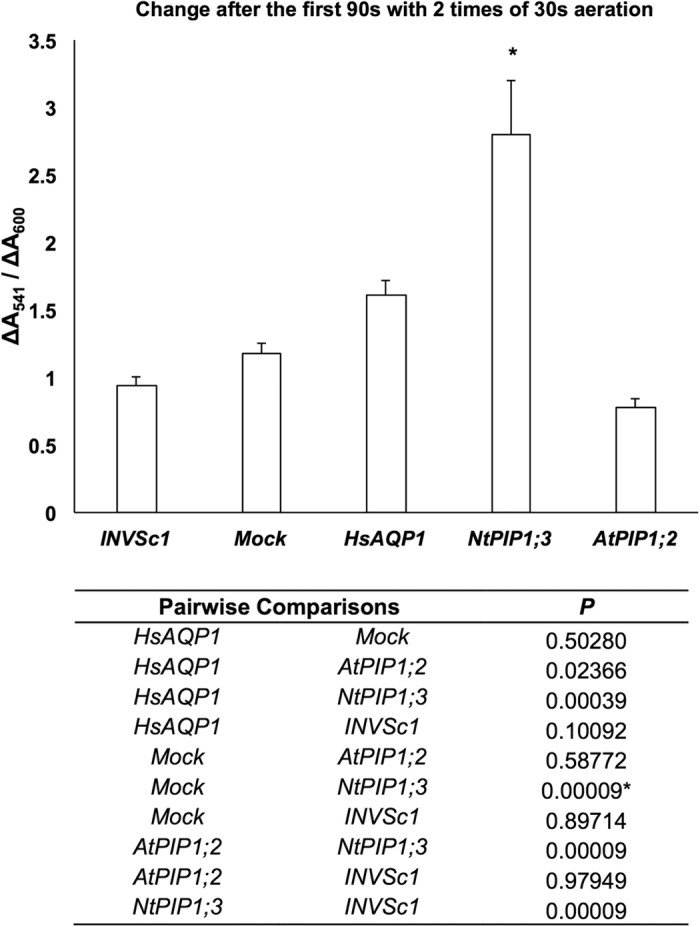
∆A_541_/∆A_600_ of yeast protoplasts after the first 90 s with 2 times of 30 s aeration. Asterisks indicate statistically significant difference with the mock strain (*P* values shown in the table below) (ANOVA, Tukey’s test, *P* ≤ 0.05, *n* = 19 ± SE).

**Figure 4:**
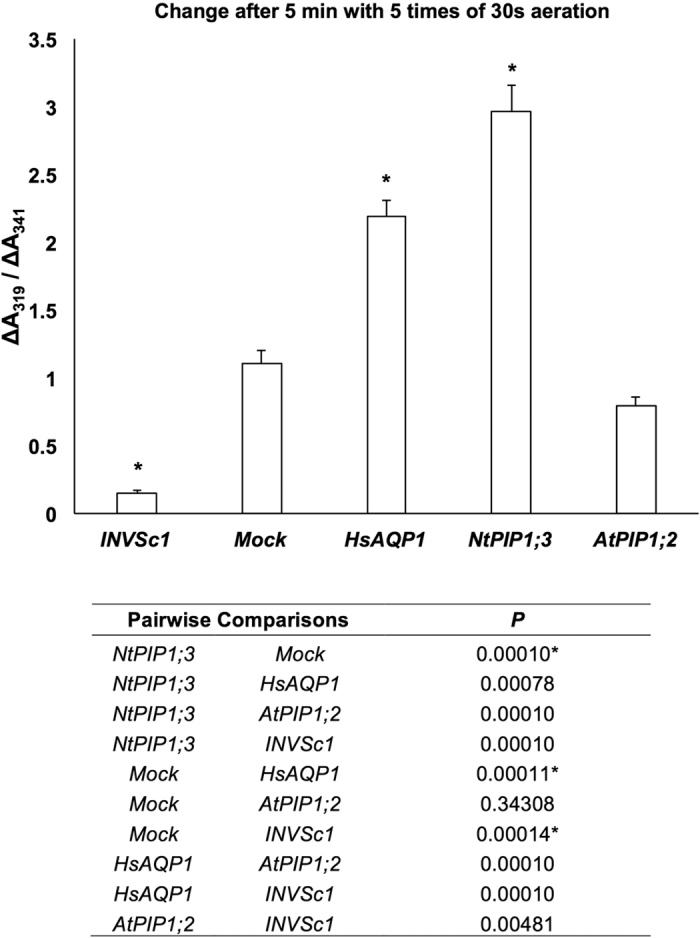
∆A_319_/∆A_341_ of yeast protoplasts after 5 min with 5 times of 30 s aeration. Asterisks indicate statistically significant difference with the mock strain (*P* values shown in the table below) (ANOVA, Tukey’s test, *P* ≤ 0.05, *n* = 6 ± SE).

Two of the most promising O_2_-transporters (HsAPQ1 and NtPIP1;3) and one that did not show O_2_-transporting properties in preliminary experiments (AtPIP1;2), as well as the mock strain were further analyzed in yeast protoplast assay. Based on the spectrum scanning on purified myoglobin (Fig. S2) and yeast protoplasts (Fig. S4), ∆A_541_/∆A_600_ and ∆A_319_/∆A_341_ (Fig. S5) were chosen to indicate myoglobin oxygenation. ∆A_541_/∆A_600_ at 90 s showed the same trend across the strains with the preliminary assay: the strain expressing NtPIP1;3 had the highest value, followed by HsAQP1, mock and AtPIP1;2 in order (Fig. 3). ∆A_319_/∆A_341_ after 5 min with 5 times of 30 s aeration demonstrated more distinct statistical difference between HsAQP1 and mock, and between all of the myoglobin-expressing strains and untransformed strain INVSc1 (Fig. 4).

Since the conversion of deoxymyoglobin to oxymyoglobin is iron-dependent, and may be affected by the cell redox status, the redox state of selected strains after being pretreated for O_2_ transport assay was measured using CM-H_2_DCFDA. Fluorescence intensity generated by CM-H_2_DCFDA showed no significant difference between yeast strains after the pretreatment of O_2_ transport assay (Fig. S6). This suggested that the cell redox state in the mock, HsAQP1, NtPIP1;3 and AtPIP1;2 strains was similar prior to the O_2_ transport assay. Similarly to the earlier report^6^, increased H_2_O_2_ permeability was detected in NtPIP1;2 strain (Fig. S7). However, no increase in H_2_O_2_ permeability was measured in NtPIP1;3, HsAQP1 or mock strains, whereas a slightly higher fluorescence intensity suggesting increased H_2_O_2_ permeability in AtPIP1;2 strain was not statistically significant (Fig. S7).

### Yeast O_2_ consumption capacity

**Figure 5:**
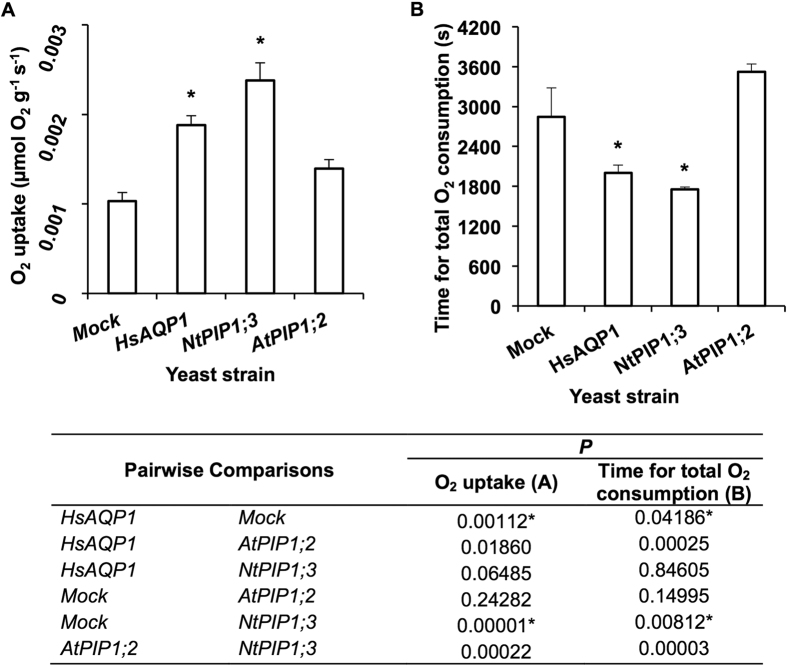
O_2_ consumption of yeast strains over 1000 s. (**A**) Respiration rates in HsAQP1, NtPIP1;3, AtPIP1;2, and mock strain (control). (**B**) Time for total O_2_ consumption in HsAQP1, NtPIP1;3, AtPIP1;2, and mock strain (control). Immediately after air was supplied to the yeast suspension in N_2_-bubbled SD-L-U + glucose medium to reach the saturation concentration of soluble O_2_ of 235 μmol L^−1^, the decrease of O_2_ concentration in yeast suspension was monitored and logged per second using an O_2_ microsensor. Asterisks indicate statistically significant difference with the mock strain (*P* values shown in the table below) (ANOVA, Tukey’s test, *P* ≤ 0.05, *n* = 6 ± SE).

Yeast cells heterologously expressing NtPIP1;3 and HsAQP1 showed 2.3-fold and 1.8-fold higher O_2_ uptake rates, respectively, compared with mock control (Fig. 5A) and depleted oxygen from the solution significantly faster (*P* ≤ 0.05) (Figs 5B and S8). The O_2_ uptake rates of yeast cells expressing AtPIP1;2 and the time for O_2_ depletion from the solution were not significantly (*P* ≥ 0.05) different from the mock controls (Fig. 5). Yeast cell diameter was not significantly affected by the heterologous expression of aquaporins and measured 2.87 ± 0.05, 2.74 ± 0.06, 2.98 ± 0.06, and 2.87 ± 0.08 μm (mean, *n* = 50 ± SE) in mock, HsAQP1, NtPIP1;3, and AtPIP1;2 strains.

### *PIP* transcript abundance and ATP level in tobacco roots under hypoxia

**Figure 6:**
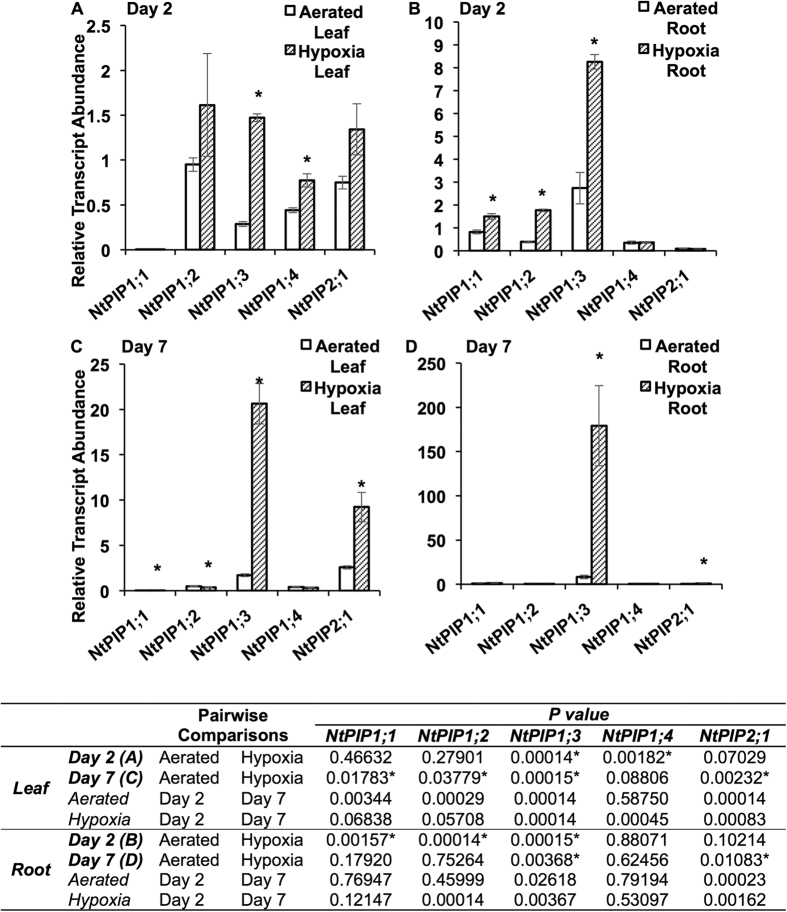
Transcript abundance of tobacco plasma membrane intrinsic proteins (*PIP*s) after exposure to well-aerated and hypoxic conditions. Relative transcript abundance of selected *PIP*s in (**A**) leaves and (**B**) roots after 2 days of exposure, and in (**C**) leaves and (**D**) roots after 7 days of exposure. Transcript abundance of *PIP*s was measured by the standard curve method in qRT-PCR assay, with normalization against geometric mean of that of the two reference genes, *EF1-α* and *L25*. Asterisks indicate significance difference in gene expression between well-aerated and hypoxic treatments on the same day; *P* values for the comparisons between day two and day seven are listed in the table below (ANOVA, Tukey’s test, *P* ≤ 0.05, *n* = 6 ± SE).

**Figure 7:**
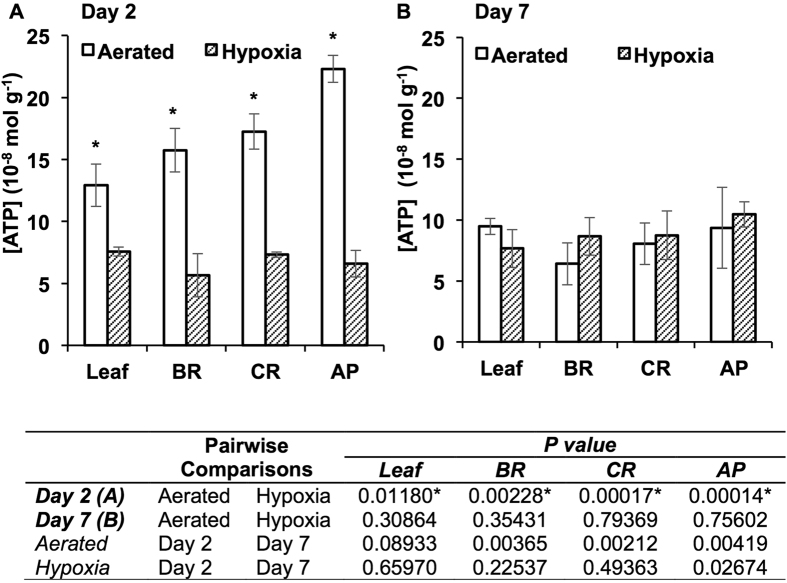
ATP levels in leaves and basal (BR), central (CR) and apical (AP) root segments of tobacco plants subjected to hypoxic and well-aerated conditions. (**A**) ATP levels after 2 days of the treatments. (**B**) ATP levels after 7 days of the treatments. ATP level was determined by detecting bioluminescence in Luciferase/Luciferin reaction. Asterisks indicate significant differences in ATP levels between well-aerated and hypoxic treatments in the same tissue on the same day; *P* values for the comparisons between day two and day seven are listed in the table below (ANOVA, Tukey’s test, *P* ≤ 0.05, *n* = 6 ± SE).

We examined transcript levels of tobacco plants subjected to flooding-induced hypoxia in mineral solution culture for two and seven days. After two days of hypoxia (≈125 μmol L^−1^ O_2_), leaf and root transcript levels of *NtPIP1*;*3* (the aquaporin showing the high rate of O_2_ transport) increased by about four-fold compared with well-aerated (≈500 μmol L^−1^ O_2_) plants (Fig. 6A,B). In well-aerated plants, aquaporin transcript levels remained similar on days two and seven in leaves and roots (Fig. 6A–D). Relatively minor increases were also measured for transcript levels of *NtPIP1*;*4* in leaves and *NtPIP1*;*1* and *NtPIP1*;*2* in roots (Fig. 6A,B). After seven days, a sharp increase of *NtPIP1*;*3* was measured in hypoxic leaves (about 12-fold higher than aerated control) and roots (about 22-fold higher than aerated control) (Fig. 6C,D). There was also about three-fold increase in *NtPIP2*;*1* in the leaves (Fig. 6C). Under the hypoxia treatment, from day two to day seven, the *NtPIP1*;*3* transcript levels sharply increased in both leaves (*P* = 0.0001) and roots (*P* = 0.0037) (Fig. 6), which was accompanied by a significant increase in ATP levels in the apical root segments (Fig. 7; *P* = 0.0267). After seven days of treatment, hypoxic and well-aerated roots had similar ATP levels in each root segment (Fig. 7B). Hypoxic plants showed healthy and green appearance, without chlorosis or other visible signs of O_2_ deficiency.

## Discussion

In this study, we investigated the potential contribution of aquaporins to transmembrane O_2_ transport in yeast whole cells and yeast protoplasts by measuring absorbance near the peak wavelengths of myoglobin over time. In the whole-cell assay, A_541_ increased over the first 60 s, which enabled us to screen strains that expressed putative O_2_-transporting aquaporins (Fig. S3B). Changes in A_541_ likely represent a combination of several processes including O_2_ diffusion, oxygenation of deoxymyoglobin, conversion between oxymyoglobin and metmyoglobin, and O_2_ consumption. The presence of cell walls might hinder the changes in absorbance in myoglobin and lead to an underestimation of O_2_ diffusion in the preliminary screening. In addition, possible artifacts on absorbance reading might be caused during the mixing of the yeast suspension and aerated buffer. The yeast protoplast assay aimed to eliminate these potential pitfalls with numerous precautions and more replications and to maximize the signal of myoglobin oxygenation. The results suggested that ∆A_319_/∆A_341_ in yeast protoplast assay may also be a highly sensitive parameter in measuring O_2_ transport through aquaporins.

Human aquaporin HsAQP1, which we found to enhance myoglobin oxygenation by facilitating O_2_ passage, was also reported to facilitate CO_2_ transport when heterologously expressed in *Xenopus laevis* oocytes^15^. However, other major CO_2_-transporting aquaporins including AtPIP1;2^5^, NtPIP1;2^3^ and LbAQP1^6^ did not facilitate O_2_ transport when expressed in yeast (Fig. S3B). This suggests that aquaporin orthologues have developed certain degree of specificity for O_2_ transport. The alignment of all the 20 analyzed aquaporins does not show consensus residues that are exclusive to O_2_-transporting aquaporins (Note S3). It appears that the conserved residues are species-dependent rather than being relevant to transport capacity. However, it is noteworthy that all six O_2_-transporting aquaporins have well-conserved 29 amino acid residues across species, including most of the Asn-Pro-Ala (NPA) signature motifs and the selective filters of Ar/R residues (Note S4). In NtPIP1;3, the asparagine residue commonly in the second NPA motif is substituted by threonine (Thr-235), which may be potentially relevant to its highly enhanced O_2_-transporting capacity.

Calculations of permeation of hydrophobic gases (O_2_, CO_2_, and NO) have consistently shown similar values of an energy barrier of 5–6 kcal mol^−1^ through water pores^16^,^17^. Membrane protein simulation systems of the human HsAQP1 tetramer have demonstrated the presence of a pore located in the center between the four monomers that is lined by largely hydrophobic residues and may be involved in the transport of gases rather than water^16^,^18^. It could be speculated that the presence and the exact structure of this pore imparts gas transport specificity to different aquaporins. In proven correct, the gating properties of this pore could be targeted to alter rates of the transmembrane passage of gases.

The results of yeast O_2_ uptake rate corroborate those of the O_2_ transport assays, pointing to the significance of pore-mediated transport for respiration. Increased transcript levels of *HsAQP1*, also sometimes accompanied by other aquaporins, have been commonly reported for cancerous cells^19^,^20^, with the level of *HsAQP1* expression often correlated with cell growth, grade of tumor^20^,^21^, and metastasis^22^,^23^. It has been also reported that the deletion of *HsAQP1* was effective in reducing breast tumor size and lung metastasis^23^ and *HsAQP1* silencing inhibited the proliferation and invasiveness of osteosarcoma cells^24^. Although the proposed explanations for the links between *HsAQP1* expression and cancerous growth have largely focused on water transport, the association between high O_2_ demand of rapidly growing cancerous cells and facilitation of O_2_ transport by HsAQP1 should also be considered.

Since the reports of hypoxia-induced expression of *HsAQP1*^25^,^26^ suggest that aquaporin-mediated transport processes may be especially important under low-O_2_ conditions, we examined transcript levels of *N. tabacum* plants subjected to flooding-induced hypoxia. Although the ATP levels showed some decline in well-aerated plants after 7 days compared with 2 days (Fig. 7A,B), the reverse trend was observed in plants subjected to root hypoxia resulting in similar ATP levels in leaves and all root segments of hypoxic and well-aerated plants after 7 days of hypoxia (Fig. 7A,B). The results suggest that after the initial hypoxic stress, plants likely received sufficient oxygen to support aerobic respiration, as hypoxic plants had healthy and green appearance and did not show chlorosis or other visible signs of O_2_ deficiency. While the resistance to root hypoxia can be explained in some plants by an increased supply of O_2_ to the root cells through the development of specialized aerating structures such as aerenchyma, the processes of plant resistance to hypoxia in the absence of obvious structural changes remain obscure. In our study, there were no structural features present in the roots and stems of plants exposed to root hypoxia that could be indicative of improved O_2_ delivery. Therefore, the increase in *NtPIP1*;*3* transcript levels (Fig. 6) could be among important factors contributing to improved root aeration, similarly to the increased transcript levels of *HsAQP1* in hypoxic human tissues^25^,^26^. Clearly, the link between pore-mediated O_2_ transport and hypoxia deserves further attention.

In conclusion, our results indicate that some of the studied plant and human aquaporins are likely to be involved in O_2_ transport. Yeast cells heterologously expressing these aquaporins maintained higher O_2_ uptake rates in liquid culture and tobacco plants exhibited sharp increases in the putative O_2_-transporting aquaporin after their roots were subjected to hypoxic conditions. These increases in O_2_ transporting aquaporins after the seven-day hypoxia treatment were accompanied by increases in ATP levels in hypoxic apical root segments. The results of the study support the notion that functional significance of pore-mediated O_2_ transport should receive more attention.

## Methods

### Expression of myoglobin and aquaporins in yeast

The complete ORF of sperm whale (*Physeter macrocephalus*) myoglobin (NCBI accession number *J03566.1*) was sub-cloned from pMB413^12^ into the yeast expression vector pAG425GAL-ccdB (http://www.addgene.org/yeast-gateway/), by the Gateway technology (Invitrogen, Carlsbad, CA, USA). The complete ORFs of the 20 aquaporin genes of interest were sub-cloned from pGEM-T Easy into the yeast expression vector pAG426GAL-ccdB (http://www.addgene.org/yeast-gateway), by the same method, respectively. These genes include three animal aquaporins from *Homo sapiens* - *HsAQP1* (*DQ895575*), *HsAQP2* (*CR542024*) and *HsAQP3* (*CR541991*), 12 plant aquaporins - *NtPIP1*;*1* (*AF440271*), *NtPIP1*;*2* (*AF024511*), *NtPIP1*;*3* (*U62280*), *NtPIP1*;*4* (*DQ914525*), *NtPIP2*;*1* (*AF440272*) and *NtXIP1*;*1* (*HM475294*) from *Nicotiana tabacum*, and *AtPIP1*;*1* (*AT3G61430*), *AtPIP1*;*2* (*AT2G45960*), *AtPIP1*;*3* (*AF348574*), *AtPIP1*;*4* (*AT4G00430*), *AtPIP1*;*5* (*AT4G23400*) and *AtNIP2*;*1* (*AT2G34390*) from *Arabidopsis thaliana*, and five fungal aquaporins from *Laccaria bicolor* – *LbAQP1* (*JQ585592*), *LbAQP3* (*JQ585593*), *LbAQP5* (*JQ585594*), *LbAQP6* (*JQ585595*) and *LbAQP7* (*JQ585596*). The constructs were verified using primer GAL1 (AATATACCTCTATACTTTAACGTC) in Sanger sequencing. *Saccharomyces cerevisiae* strain INVSc1 (MATa his3D1 leu2 trp1-289 ura3-52; Invitrogen) was double-transformed with pAG425GAL-ccdB + myoglobin vector and one of the PAG426GAL-ccdB + aquaporin vectors, following the protocol of small-scale yeast transformation (Invitrogen). For mock control, INVSc1 was transformed with pAG425GAL-ccdB + myoglobin vector and empty PAG426GAL-ccdB vector.

Selection was based on ura3 and leu2 complementation. Transformed yeasts were cultured in glucose containing synthetic complete medium without Ura/Leu (United States Biological) (2 g of yeast nitrogen base, 2 g of dropout amino acids, 5 g of (NH_4_)_2_SO_4_, 30 g of glucose in 1 L of SD-L-U + glucose medium, pH = 6) for 24 h at 1.2× *g* and 30 °C. Cultures were diluted to an optical density of OD_600_ = 0.6. Heterologous protein expression was induced by changing the carbon source of the medium from glucose to galactose (30 g in 1 L of SD-L-U + galactose medium) and growing yeast cells for 24 h (1.2× *g*, 30 °C), with 25 mg L^−1^ FeSO_4_ as iron source to promote the formation of myoglobin-iron binding structure^27^, validated by transcript abundance assay of quantitative RT-PCR^28^,^29^ (Method S1), immunoblotting (Method S2) and indirect immunofluorescence detection (Method S3).

### O_2_ Transport Assay

The yeasts were washed in KH_2_PO_4_ buffer (0.1 M, pH 6) twice, and then suspended in N_2_-bubbled KH_2_PO_4_ buffer. The yeast suspension was bubbled with N_2_ for 30 s and vacuumed for 30 min, to minimize the soluble O_2_ in yeast suspension, which is crucial to maintain the state of deoxymyoglobin^30^,^31^. The spectrum between 500 nm and 600 nm of yeast suspension was scanned after O_2_ depletion and re-aeration. Compared to the spectrum of purified myoglobin^31^, the spectrum of yeast suspension suggests that the state of metmyoglobin was likely dominant over deoxymyoglobin and oxymyoglobin. In addition, the absorbance spectrum of yeast cell suspension is expectably more complex than purified myoglobin proteins. Despite these limitations, the change in A_541_ after re-aeration was noticeable (Fig. S3A), which was in the range of 541–543 nm, i.e., the absorption peak of purified oxymyoglobin in the study of Zhao *et al*.^30^ as well as in our observation (Fig. S2). The increase in A_541_ reflects the conversion of deoxymyoglobin into the oxygenated state upon Fe^2+^ - O_2_ binding, which can be attributed to O_2_ influx. The rate of increase in A_541_ (∆A_541_ s^−1^) can reflect the capacity of O_2_ uptake by the yeast strains expressing different aquaporins. Therefore, absorbance of 1 mL yeast suspension of each strain was recorded at 541 nm for 2 min at 1 s interval immediately after the addition of 1 mL of air-saturated KH_2_PO_4_ buffer or N_2_-saturated KH_2_PO_4_ as negative control, respectively, using a spectrophotometer (Thermo Genesys 10S V4.002, ThermoFisher Scientific). All measurements were carried out at 22 °C. The mean and standard error were calculated based on six biological replications. CM-H_2_DCFDA^32^ was used to indicate oxidative state of selected yeast strains due to such pretreatment and to determine H_2_O_2_ transport capacity of selected aquaporins^33^ (Methods S4).

After screening the strains that expressed putative O_2_-transporting aquaporins by measuring the increase in A_541_ over 60 s with the spectrophotometer (Fig. S3B), yeast protoplasts were prepared for the selected ones for the refined O_2_ transport assay. After induction, 4 mL of yeast culture at OD_600_ = 2 of each strain was harvested, pre-incubated, washed and treated with zymolyase (Yeast lyticase 100 T, United States Biological) at 37 °C, 50 rpm for 2 hr. Yeast protoplasts were re-suspended in 10 mL of enzyme buffer (1.2 M sorbitol, 50 mM magnesium acetate, 10 mM CaCl_2_ in autoclaved deionized distilled water). For the initial O_2_-depleted state, the absorbance spectrum was scanned from 300 nm to 650 nm immediately after mixing 500 μL of protoplast suspension with 500 μL of isosmotic sodium ascorbate buffer (0.6 M sodium ascorbate, 50 mM magnesium acetate, 10 mM CaCl_2_ in autoclaved deionized distilled water). Sequential scanning was conducted after each 30 s of direct aeration at time points of 30 s, 90 s, 150 s and every minute up to the 10^th^ min. At 541–543 nm, myoglobin of the oxygenated state has a pronounced absorbance peak^31^ (Fig. S2). At 319–330 nm, both myoglobin (Fig. S2) and myoglobin-expressing yeast protoplasts showed a second, much more pronounced absorbance peak in the oxygenated state (Fig. S4B–E), which was absent in untransformed yeast strain (Fig. S4A). The value of A_319_/A_341_ increased dramatically along with multiple aeration (Fig. S5C), suggesting ∆A_319_/∆A_341_ be a good indicator for myoglobin oxygenation. Therefore, both ∆A_541_/∆_A600_ and ∆A_319_/∆A_341_ were calculated to present the change in absorbance due to myoglobin oxygenation. Statistically significant difference across all the strains was analyzed in ∆A_541_/∆A_600_ of yeast protoplasts at 90 s (ANOVA, Tukey test, *P* ≤ 0.05, *n* = 19; *P* values shown in the table of Fig. 3) and in ∆A_319_/∆A_341_ after 5 min with 5 times of 30 s aeration (ANOVA, Tukey test, *P* ≤ 0.05, *n* = 6; *P* values are shown in the table of Fig. 4).

For spectrum scanning of myoglobin, purified horse myoglobin protein at 1 mg/mL was mixed with 1 volume of 10% sodium ascorbate to generate the deoxygenated state, followed by the above-mentioned series of aeration to achieve the state of oxymyoglobin.

### Oxygen uptake by yeast

O_2_ uptake rates in yeast suspension culture were continuously monitored over 40 min in the over-expression and mock strains (Fig. S8). The time required for the yeast suspension cultures to deplete O_2_ from the solution was also measured, with glucose as a carbon source. *S. cerevisiae* strains INVSc1 for the expression of HsAQP1, or NtPIP1;3, or AtPIP1;2, and the mock control strain, were cultured and induced for heterologous protein expression as described above. The yeasts were washed in KH_2_PO_4_ buffer (0.1 M, pH 6) twice, and then suspended in 15 mL of N_2_-bubbled SD-L-U + glucose medium in 50 mL Falcon tubes to reach OD_600_ = 5. Air was supplied into the yeast suspension until its concentration of soluble O_2_ reached about 235 μmol L^−1^, the stable saturation level of the still medium at 25 °C. Starting from this point, the decrease of O_2_ concentration in yeast suspension was monitored and logged per second using an O_2_ microsensor with tip diameter of 50 μm (OX-50) connected to the OXY-Meter, a compact O_2_ microsensor amplifier (Unisense, Aarhus, Denmark). Parafilm was used to seal and minimize free air diffusion to the Falcon tubes. The slopes of the decline in O_2_ concentration during the initial 0–1000 s were calculated by linear regression, in which absolute values represented the rates of O_2_ consumption by different yeast strains during the corresponding intervals. O_2_ depletion time of each yeast suspension was recorded. The means and standard errors were calculated based on six biological replications.

### Tobacco Root Hypoxia Study: Growth Conditions and Treatment

Tobacco *(Nicotiana tabacum L.)* seeds were germinated in soil and plants grown for two weeks in a controlled-environment growth room maintained at 22/18 °C (day/night) temperatures, 60 ± 10% relative humidity, and 18-h photoperiod with photosynthetic photon flux density of approximately 350 μmol m^−2^ s^−1^. After two weeks of growth, plants were transferred to containers with half-strength modified Hoagland’s solution aerated with aquarium pumps (dissolved O_2_ of approximately 7.5 mg L^−1^). Thirty-six plants were randomly placed in six containers. After one week, the plants in three containers were subjected to hypoxia by flushing water with nitrogen gas to reach a dissolved O_2_ level of 2 mg L^−1^, and then left stagnant. The plants in the other three containers continued to be aerated.

### Quantitative RT-PCR in tobacco

Roots and leaves were sampled after two and seven days of hypoxia treatment (*n* = 6). The tissue samples were frozen and homogenized in liquid nitrogen using mortar and pestle. Total RNA was extracted using RNeasy Plant Mini Kit (Qiagen, Valencia, CA, USA). The cDNA synthesis and qPCR were conducted as described in Method S1. The transcript abundance of *NtPIP1*;*1, PIP1*;*2, PIP1*;*3, PIP1*;*4* and *PIP2*;*1* was normalized against geometric mean of that of the two reference genes, *EF1-α* and ribosomal protein *L25*. Gene-specific primers were designed using Primer Express 3.0 (Applied Biosystems, Life Technologies) (Table S2).

### Determination of ATP Level

ATP levels were measured in leaves and apical, central and basal root segments after 2 and 7 days of hypoxia and in well-aerated plants. Roots and leaves of tobacco were sampled after two and seven days of treatments. The roots were divided into the basal, apical and central segments. Tissue samples were ground and 50 mg ground samples were placed in 600 μL of ice-cold 5% Trichloroacetic acid (TCA)^34^ in 2 mL centrifuge tubes. The samples were vigorously vortexed for 20 s, left on ice for 10 min and centrifuged at 10,000× *g*, 4 °C for 10 min. Each 400 μL of supernatant was collected and added to 400 μL of ice-cold Tris-acetate buffer (pH = 7.75, 1 M). For the ATP assay, 4 μL of the mixture was pipetted into 96 μL of ATP-free water into a well of the 96-well plate (Costar 96 well plate with flat bottom). To quantify ATP, 50 μL of rLuciferase/Luciferin reagent from ENLITEN ATP Assay Kit (Promega, Madison, WI, USA) was added into each well, and the standard curve was prepared following the manufacturer’s protocol. Bioluminescence signal^35^ was detected using a microplate reader (Fluostar Optima, BMG Labtech, Ortenberg, Germany).

### Statistical Analysis

The means and standard errors were calculated based on the biological replications in each assay by descriptive statistics. Statistical difference was analyzed using one-way ANOVA (Tukey’s test, *P* ≤ 0.05).

## Additional Information

**How to cite this article**: Zwiazek, J. J. *et al*. Significance of oxygen transport through aquaporins. *Sci. Rep.* **7**, 40411; doi: 10.1038/srep40411 (2017).

**Publisher's note:** Springer Nature remains neutral with regard to jurisdictional claims in published maps and institutional affiliations.

## Supplementary Material

Supplementary Information
