# Supplementary material for: Significance of oxygen transport through aquaporins
Source: Sci Rep. 2017 Jan 12;7:40411. doi: 10.1038/srep40411 (PMC5227684; doi:10.1038/srep40411)
Supplement: Supplementary Information [file srep40411-s1.doc]

# Supplementary Information

# Significance of oxygen transport through aquaporins

Janusz J. Zwiazek,^1^* Hao Xu,^1^ Xiangfeng Tan,^1^ Alfonso Navarro-Ródenas,^1,2^ Asunción Morte^2^

^1^Department of Renewable Resources, University of Alberta, Edmonton, AB, T6G 2E3, Canada.

^2^Departamento de Biología Vegetal (Botánica), Facultad de Biología, Universidad de Murcia, Campus de Espinardo, 30100 Murcia, Spain.

*Corresponding author. Email: [jzwiazek@ualberta.ca](mailto:jzwiazek@ualberta.ca)

Supplementary Information

Supplementary Methods [3](#__RefHeading___Toc337117193)

Methods S1 Quantitative RT-PCR in yeast [3](#__RefHeading___Toc337117194)

Methods S2 SDS-PAGE and immunoblotting [3](#__RefHeading___Toc337117195)

Methods S3 Indirect immunofluorescence [4](#__RefHeading___Toc337117196)

Methods S4 Oxidative state and H_2_O_2_ Transport Assay [6](#__RefHeading___Toc337117197)

Supplementary Figures [9](#__RefHeading___Toc337117198)

Fig. S1 Transcript level of myoglobin and aquaporins in the transformed yeast strains. [9](#__RefHeading___Toc337117199)

Fig. S2 Effect of aeration on absorbance spectra of purified myoglobin protein. [10](#__RefHeading___Toc337117200)

Fig. S3 The expression of O_2_-transporting aquaporins in yeasts led to higher rate of increase in A_541_ of cell suspension due to oxygenation of the co-expressed myoglobin. [11](#__RefHeading___Toc337117201)

Fig. S4 Scanning of absorbance spectra of yeast protoplasts from 300 nm to 650 nm during 10 min with multiple times of aeration. [13](#__RefHeading___Toc337117202)

Fig. S5 Change in A_541_, A_319_ and A_319_/A_341_ of yeast protoplasts during 10 min with multiple times of aeration. [14](#__RefHeading___Toc337117203)

Fig. S6 Cellular oxidative stress in yeast strains after pre-treatment for O_2_ transport assay using CM-H_2_DCFDA as the ROS indicator. [15](#__RefHeading___Toc337117204)

Fig. S7 Effect of the expression of NtPIP1;2 (NtAQP1), NtPIP1;3, HsAQP1, AtPIP1;2, and the mock control of empty expression vector on H_2_O_2_ permeability of yeast plasma membrane. [16](#__RefHeading___Toc337117205)

Fig. S8 Characteristics of O_2_ consumption of yeast strains. [17](#__RefHeading___Toc337117206)

Supplementary Tables [18](#__RefHeading___Toc337117207)

Table S1 Subcellular localization of expressed aquaporin proteins predicted by TargetP 1.1 server [18](#__RefHeading___Toc337117208)

Table S2 The qPCR primers for gene transcript abundance assays in *Saccharomyces cerevisiae* and *Nicotiana* *tabacum* [18](#__RefHeading___Toc337117209)

Supplementary Notes [19](#__RefHeading___Toc337117210)

Note S1 Accession numbers and full-length open reading frames of the 20 aquaporin genes in this study [19](#__RefHeading___Toc337117211)

Note S2 Amino acid sequences of aquaporins in this study deduced by NCBI ORF Finder [25](#__RefHeading___Toc337117212)

Note S3 Alignment of amino acid sequences of the aquaporins in this study by CLUSTAL O (V.1.2.1) [28](#__RefHeading___Toc337117213)

Note S4 Alignment of amino acid sequences of six O_2_-transporting aquaporins by CLUSTAL O (V.1.2.1) [31](#__RefHeading___Toc337117214)

# Supplementary Methods

## Methods S1 Quantitative RT-PCR in yeast

After 24-h induction in SD-L-U galactose solution, five milliliters of cell suspension at OD_600_ = 1 were harvested at 3000 *× g* for 5 min and frozen in liquid N_2_ for cell lysis and RNA extraction by Trizol® method (Thermofisher, manufacturer’s protocol) (*n* = 3). The first strand cDNA was synthesized from 1 μg total RNA using QuantiTect Reverse Transcription Kit (Qiagen), and used for SYBR Green™ quantitative real-time polymerase chain reaction (qRT-PCR) with standard curve method. Each 10 μL of the reaction consisted of 2.5 μL of 1.6 μM primer, 2.5 μL of 10 ng/μL cDNA and 5 μL of qPCR Mastermix^28^. The transcript abundance of myoglobin, *HsAQP1*, *NtPIP1;3* and *AtPIP1;2* (Table S2) in the transformed strains was normalized to the yeast reference gene *ScFBA1* (YKL060C)^29^, by relative quantification of standard curve method with correction for amplification efficiency.

## Methods S2 SDS-PAGE and immunoblotting

After induction, 20 mL of *S. cerevisiae* culture (OD_600_ = 2.5) was centrifuged at 600 *× g* for 5 min to yield 120 mg fresh weight yeast cells for total protein extraction (Kushnirov 2000). The pellet was suspended in 500 µL of distilled water and 500 µL of 0.2 M NaOH was added followed by incubation for 5 min at room temperature. Following centrifugation, the resulting pellet was, re-suspended in 250 µL of SDS sample buffer, boiled for 3 min and pelleted again. The protein-containing supernatant was stored at -20 °C for future use.

About 15 µg of extracted total protein in sample buffer was loaded in each sample lane of the SDS-PAGE precast gel (Mini-PROTEAN TGX Precast Gel, Bio-Rad, Berkeley, CA, USA), including the mock strain that expressed whale myoglobin but no heterologous aquaporin, yeast strains that expressed whale myoglobin and aquaporin HsAQP1, NtPIP1;3 or AtPIP1;2, respectively, and untransformed yeast strain INVSc1. Three microliters of protein standard (Precision Plus Protein DualColor Standards, Bio-Rad) was loaded in the ladder lane. Two micrograms of 17-kDa purified horse myoglobin (Sigma-Aldrich, St Louis, MO, USA) was used as the positive control. The gels were stained with Commassie Blue or transferred onto PVDF membrane, blocked in 3% BSA in TBST at 4 °C for 12 h, and incubated in the solution of 1:500 diluted primary polyclonal anti-myoglobin antibody produced in rabbit (Sigma-Aldrich) overnight at 4 °C. The western blots were then incubated in 1:3000 diluted secondary antibody of GAR-alkaline phosphatase (Bio-Rad) at room temperature for 1 h with gentle agitation. Color was developed for 2 min using alkaline phosphatase conjugate substrate kit (Bio-Rad).

For aquaporin detection, the blocked membrane was incubated in the solution of 1:2000 diluted primary anti-human aquaporin 1 monoclonal antibody produced in mouse (OriGene) at 4 °C overnight, followed by incubation in the solution of 1:10000 diluted secondary antibody of GAM IgG-alkaline phosphatase (Sigma-Aldrich) at room temperature for 1 h with gentle agitation. Color was developed for 1 min using alkaline phosphatase conjugate substrate kit (Bio-Rad).

## Methods S3 Indirect immunofluorescence

One milliliter of yeast suspension at OD_600_ = 1 was centrifuged at 600 *× g* for 5 min. The pellets were re-suspended in 500 µL of heated 3% agarose. The solidified agarose-yeast plugs were kept in formaldehyde-acetic acid- ethanol (FAA) for fixation in vacuum for 24 h. The samples were embedded in paraffin and sliced using a microtome. The sections were kept at 37 °C overnight, de-waxed in toluene, rehydrated, and circled out using an ImmEdge® hydrophobic barrier pen, before blocking and antibody incubation in a humidity chamber. The cells in the circle were blocked by adding a drop of PBS buffer with 2% of BSA and 0.1% of Tween-20 at room temperature for 5 min. The blocked yeasts were incubated in the solution of 1:100 diluted primary anti-aquaporin 1 monoclonal antibody produced in mouse (OriGene) at 4 °C overnight, followed by incubation in the solution of 1:50 diluted secondary antibody of Fluorescein (FITC-isomer 1; A max = 492 nm, E max = 520 nm)-conjugated AffiniPure rat anti-mouse IgG (H+L) (Jackson ImmunoResearch) at room temperature in dark for 2 h. The yeast cells of mock strain that had not been treated with the primary antibody were the negative control to show the background of autofluorescence. The cells were washed in blocking buffer for 3 times. A drop of water was added onto the samples before mounting the slide cover. The green fluorescence was viewed under 40× or 100× objective lens of a Leica HC fluorescence microscope with blue light excitation. The images were captured using Q-Capture Pro 7, with exposure time as 2650 ms for 40× objective lens and 100 ms for 100× objective lens. Subcellular localization of the aquaporins was also predicted using TargetP1.1^14^, based on the predicted presence of any of the N-terminal presequences, mitochondrial targeting peptide or secretory pathway signal peptide in eukaryotic cells.

In this assay assay, the whole yeast cells had gone through the process of FAA fixing, paraffin-embedding, dewaxing and microtome slicing, before the antibody binding for immunofluorescence detection. The primary antibody affinity to the epitopes of HsAQP1 might have been dramatically hindered by this process and also by the presence of cell walls, leading to the strongest fluorescence signals being observed on the periphery of the cross-sectioned yeast cell – where the plasma membrane had the most exposure to the antibody. The fluorescence microscope that we used for observation was not confocal, therefore we could not demonstrate the fluorescence signals emitted from multi-panels simultaneously. Particularly under 100X objective lens, the focus could only reveal the fluorescence emitted within a very limited depth, which caused one of the two cells in Panel B of Fig. 2 not to show in the Panel A. However, under 40X objective lens, the fluorescence from both cells was visible (Fig. Methods S3-1). We also conducted direct immunofluorescence assay on the yeast protoplast. It showed relatively evenly distributed fluorescence over the surface of protoplasts of HsAQP1 yeast strain (Fig. Methods S3-2, 40X), probably due to higher antibody binding affinity caused by the absence of cell wall or no interference of the embedding process. It indicated the plasma membrane of the yeast protoplast as the likely localization of HsAQP1; meanwhile, it did not exclude the possibility of its intracellular localization.


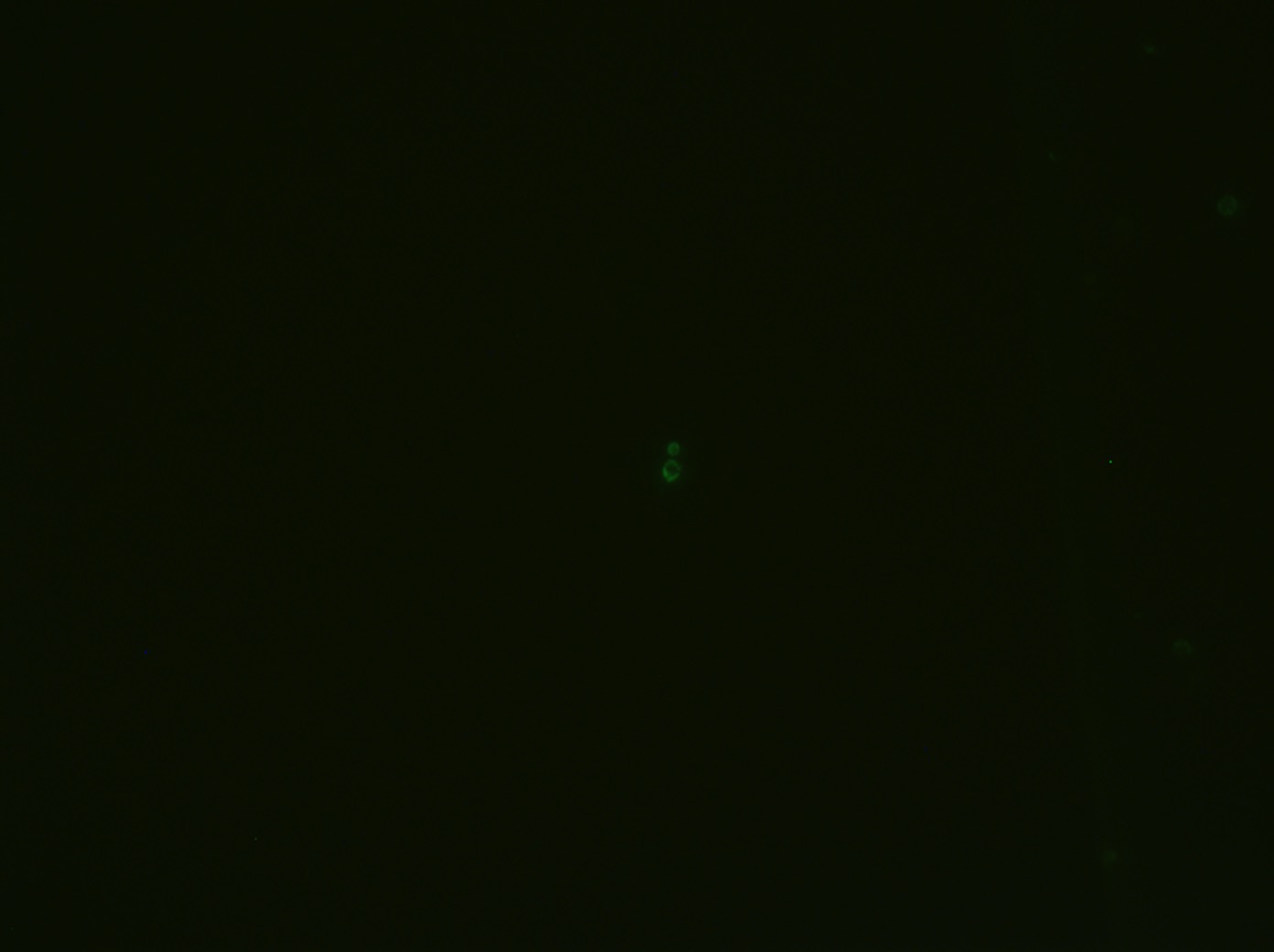

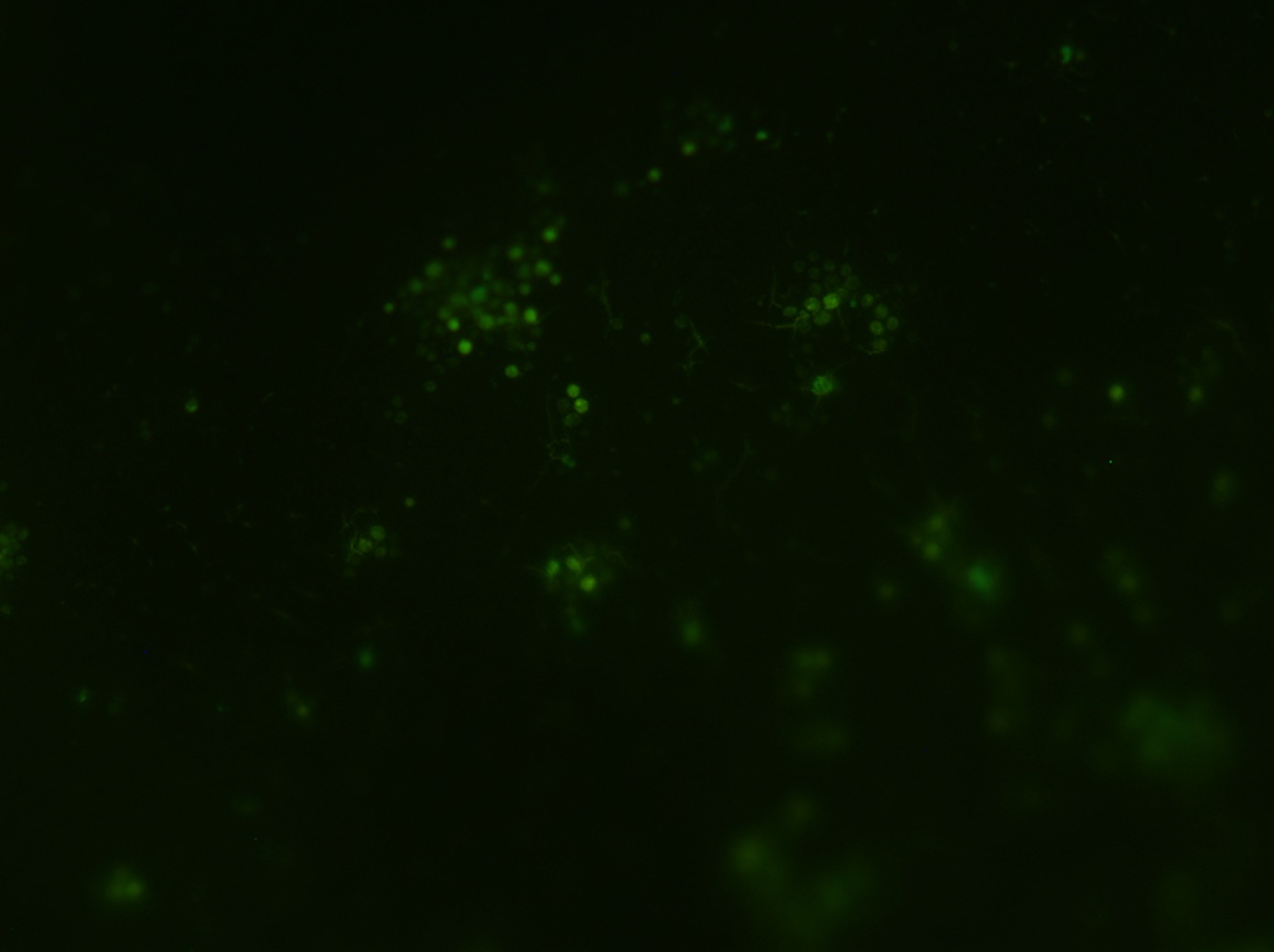


Fig. Methods S3-1

Indirect immunofluorescence of paraffin-embedded, microtome sliced yeast cells under 40X objective lens. Bar is 5 µm .

Fig. Methods S3-2

Direct immunofluorescence of yeast protoplasts under 40X objective lens. Bar is 5 µm .

##

## Methods S4 Oxidative state and H_2_O_2_ Transport Assay

CM-H_2_DCFDA, the derivative of H_2_DCFDA, one of the most widely used methods for directly measuring the redox state in cells ^32^, was used to indicate the redox state and the oxidative stress in selected yeast strains that had been pretreated for O_2_ transport assay. NtPIP1;2 strain was used as a positive control since this aquaporin (i.e., NtAQP1) was previously demonstrated to transport H_2_O_2_ ^3, 6^. Heterologous protein expression was induced in the SD-L-U + galactose medium, for 24 h in dark (1.2 *× g*, 30 °C), with 25 mg L^-1^ of FeSO_4_ as iron source and 1 µM of CM-H_2_DCFDA as the ROS indicator. The yeasts were washed twice with KH_2_PO_4_ buffer (0.1 M, pH 6) and then suspended in N_2_-bubbled KH_2_PO_4_ buffer. The yeast suspension was bubbled with N_2_ for 30 s and vacuum was applied for 30 min. Fluorescence generated from each 200 µL sample was recorded using a microplate reader (Fluostar Optima, BMG Labtech, Ortenberg, Germany) with excitation wavelength of 485 nm and emission wavelength of 520 nm at 27 s intervals for 10 min (*n* = 6).

Strain of *S. cerevisiae* INVSc1 (MATa his3D1 leu2 trp1-289 ura3-52; Invitrogen) containing the empty pAG425GAL-ccdB vector was transformed with the vector pAG426GAL-ccdB expressing *Homo sapiens HsAQP1* (P29972), *Nicotiana tabacum NtPIP1;2 (NtAQP1*, AJ001416) and *NtPIP1;3* (U62280), *Arabidopsis thaliana AtPIP1;2* (AT2G45960) or the empty vector pAG426GAL-ccdB as the mock control, respectively. Transformed yeasts were cultured in glucose containing synthetic complete medium without Ura/Leu for 24 h (1.2 *× g*, 30 °C). Cultures were diluted to OD_600_ = 0.6, and heterologous protein expression was induced by changing the carbon source of the medium from glucose to galactose. CM-H_2_DCFDA [5-(&-6)-chloromethyl-2, 7-dichlorodihydrofluorescein diacetate, acetylester mixed isomers] (Life Technologies; Carlsbad, CA, USA) was used for detecting intracellular H_2_O_2_ and as an indicator to measure the effect of aquaporin expression on H_2_O_2_ permeability of yeast cells ^33^. Greater increase in fluorescence over time indicates higher intracellular concentration of the examined molecule H_2_O_2_ being transported.

Yeasts were grown and dyed for 24 h in dark (1.2 *× g*, 30 °C) in galactose synthetic medium with 1 µM of CM-H_2_DCFDA, and then were washed with 20 mM HEPES buffer (pH = 7) for five times and re-suspended in HEPES to reach OD_600_ = 1.4 ^6^. Non-dye mock yeasts were examined to show the background fluorescence. In H_2_O_2_ treatment, 100 µM H_2_O_2_ was used as H_2_O_2_ source. In H_2_O_2_ + AgNO_3_ treatment, yeasts were incubated in 15 µm of the aquaporin inhibitor AgNO_3_ for one hour in dark before H_2_O_2_ was added. In HEPES treatment, neither H_2_O_2_ nor AgNO_3_ was applied. Fluorescence generated from each 200 µL reaction per well of a 96-well microplate was recorded using a microplate reader (Fluostar Optima, BMG Labtech, Ortenberg, Germany) with excitation wavelength of 485 nm and emission wavelength of 520 nm at 27 s intervals for 30 min (*n* = 6).

# Supplementary Figures


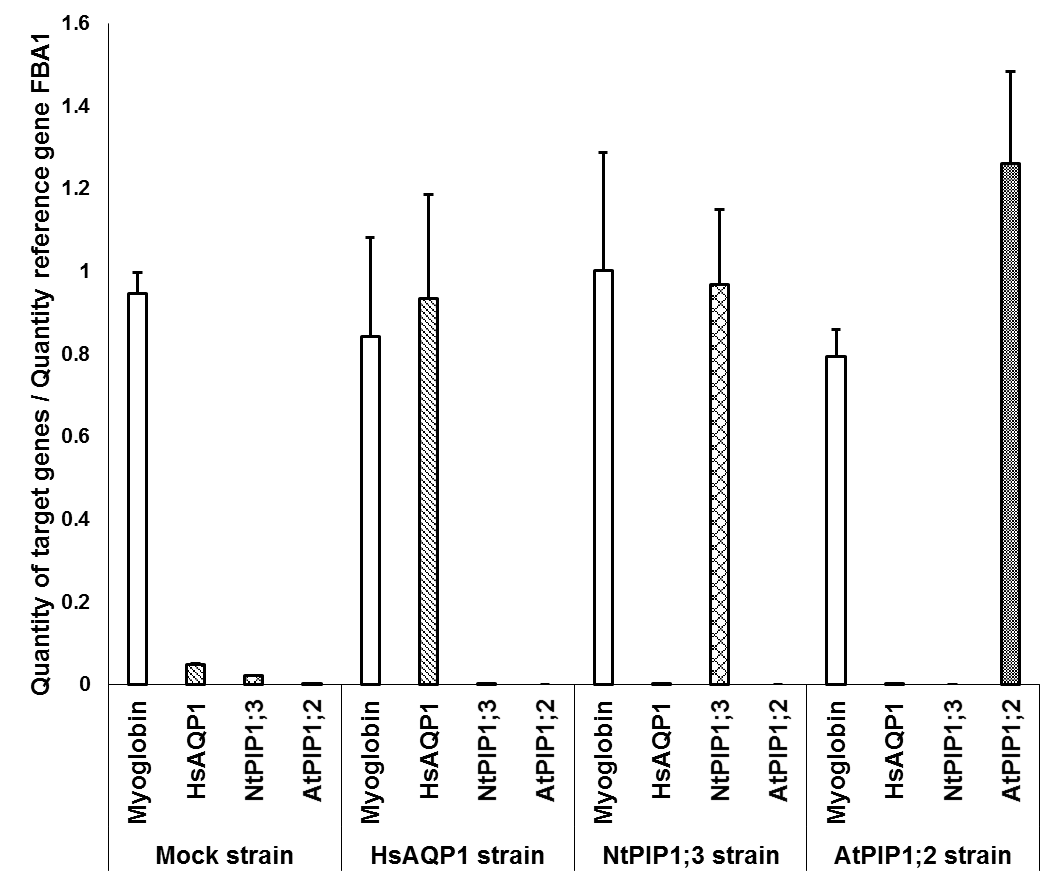


## Fig. S1 Transcript level of myoglobin and aquaporins in the transformed yeast strains.

The transcript abundance of myoglobin, *HsAQP1*, *NtPIP1;3* and *AtPIP1;2* in each strain was normalized to the yeast reference gene *ScFBA1*, by relative quantification of standard curve method with correction for amplification efficiency (*n* = 3).


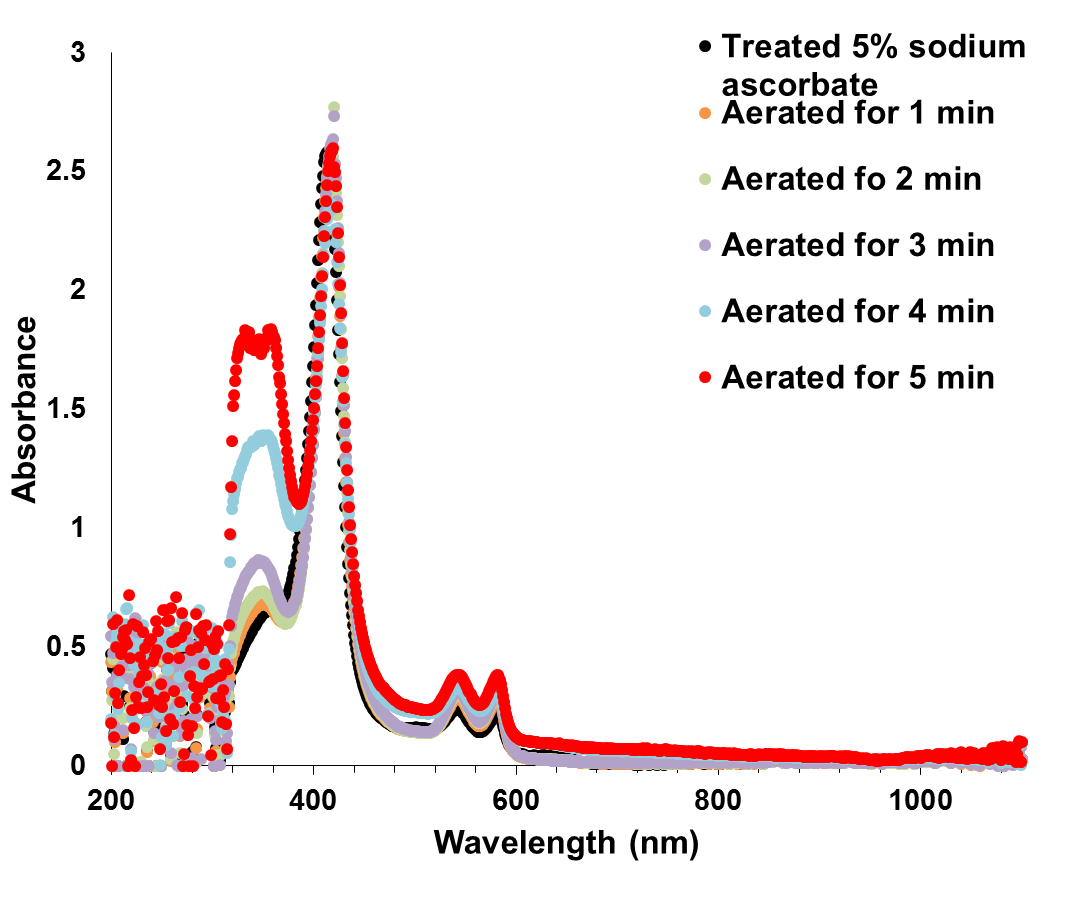

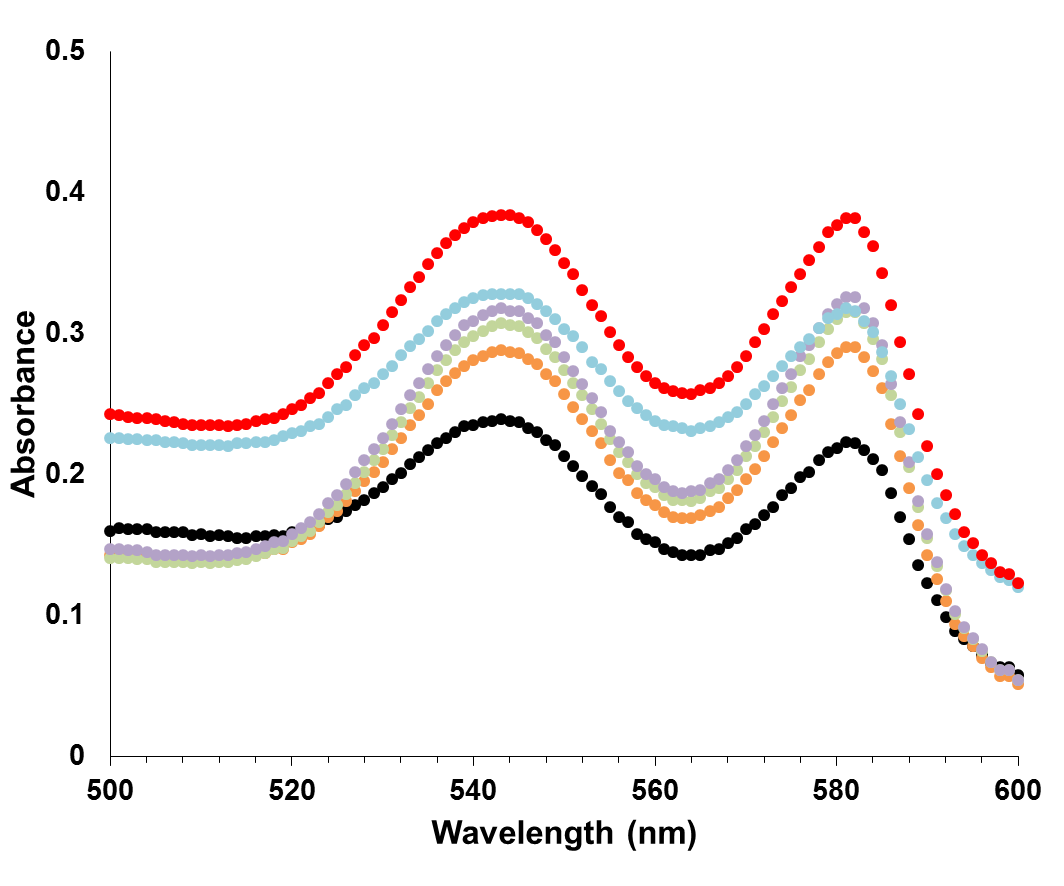


**A**

**B**

## Fig. S2 Effect of aeration on absorbance spectra of purified myoglobin protein.

(**A**) Absorbance spectrum from 190 nm to 1100 nm. (**B**) Absorbance spectrum from 500 nm to 600 nm.


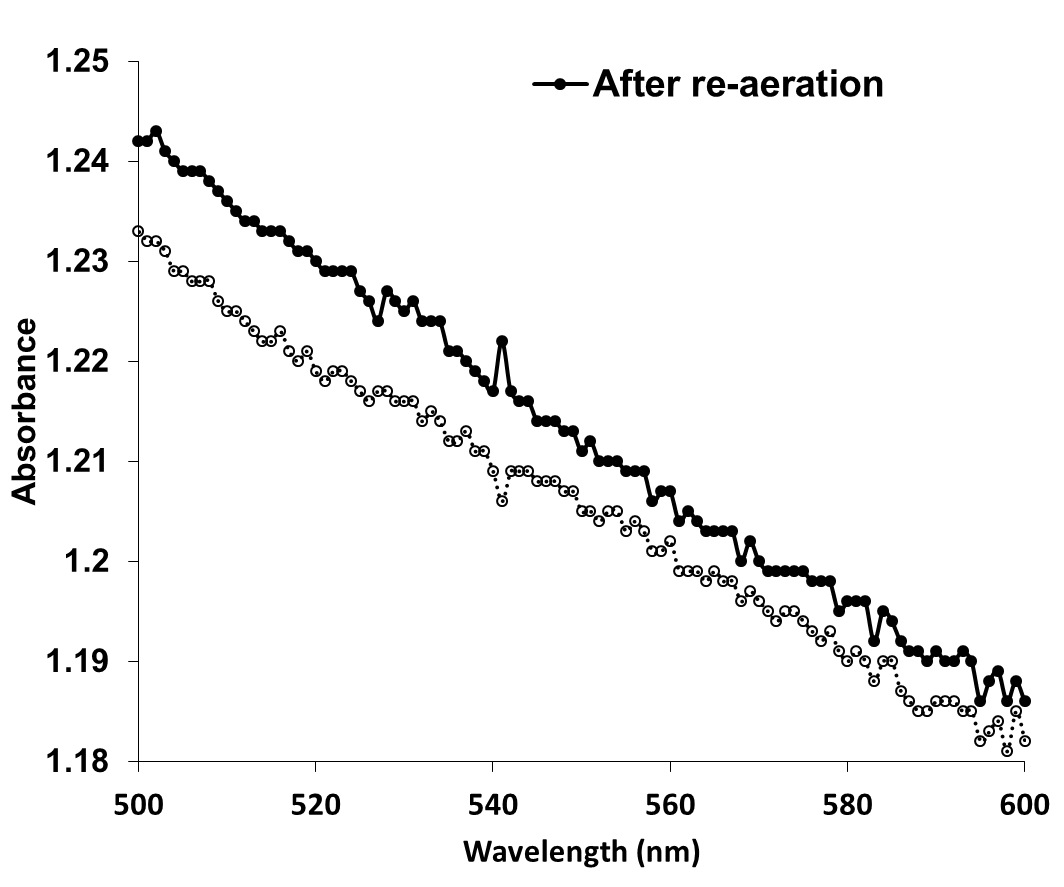

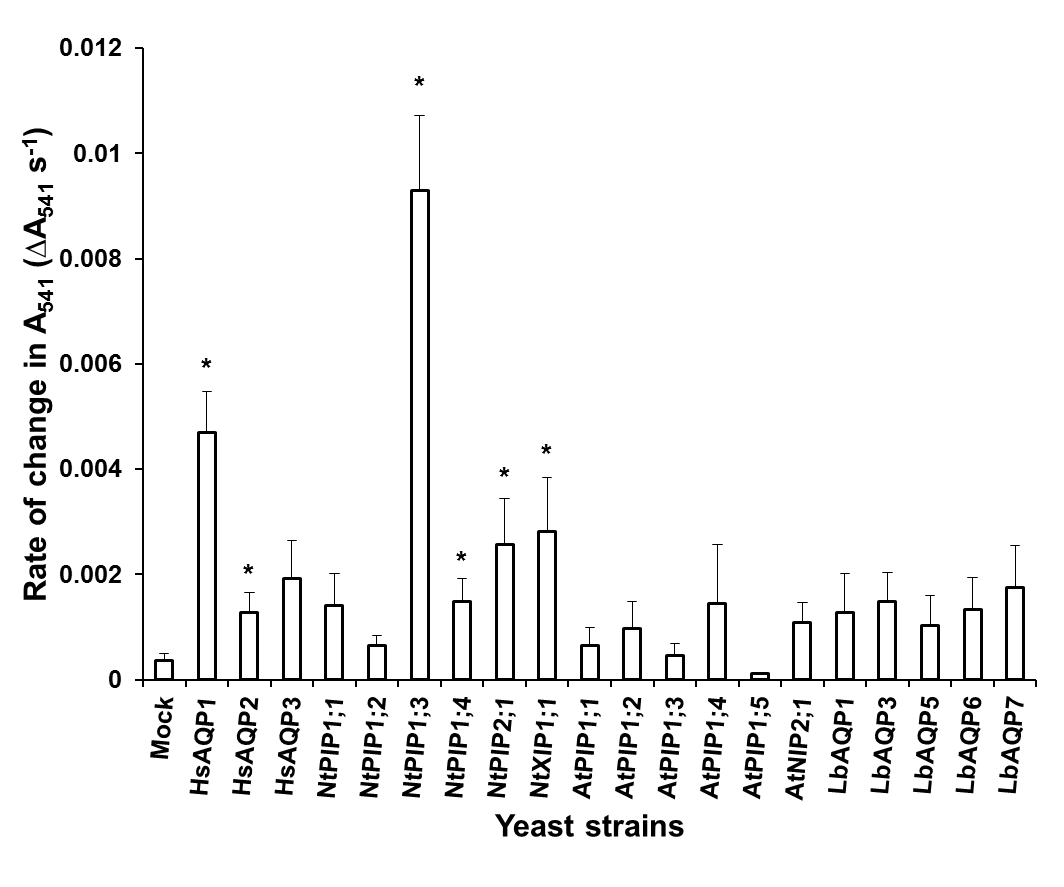


**A**

**B**

## Fig. S3 The expression of O_2_-transporting aquaporins in yeasts led to higher rate of increase in A_541_ of cell suspension due to oxygenation of the co-expressed myoglobin.

(**A**) Absorbance spectrum between 500 nm and 600 nm for HsAQP1 yeast strain after oxygen depletion or re-aeration. (B) Rate of increase in A_541_ in 20 strains of yeasts co-expressing myoglobin and different human (Hs), *Nicotiana tabacum* (Nt), *Arabidopsis thaliana* (At) and *Laccaria bicolor* (Lb) aquaporins and in a mock strain (control). The sperm whale myoglobin and one of the 20 different aquaporins were co-expressed in yeast strain INVSc1, and the rate of O_2_ uptake was reflected by the rate of increase in A_541_ (∆A_541_ s^-1^) due to myoglobin oxygenation. A_541_ of 1 mL yeast suspension of each strain was recorded for 2 min at 1 s interval immediately after the addition of 1 mL of air-saturated KH_2_PO_4_ buffer or N_2_-saturated KH_2_PO_4_ as negative control, respectively, to calculate ∆A_541_ s^-1^. The means and standard errors were calculated from six biological replications (*n* = 6 ± SE). Asterisks indicate statistically significant difference (ANOVA, Tukey’s test, P ≤ 0.05).


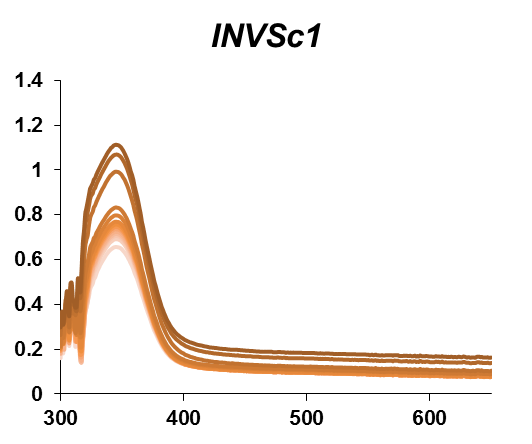

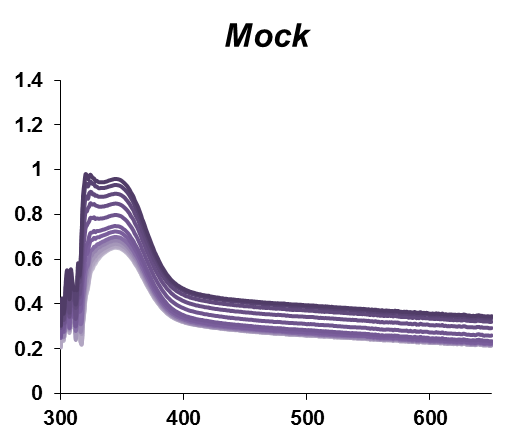

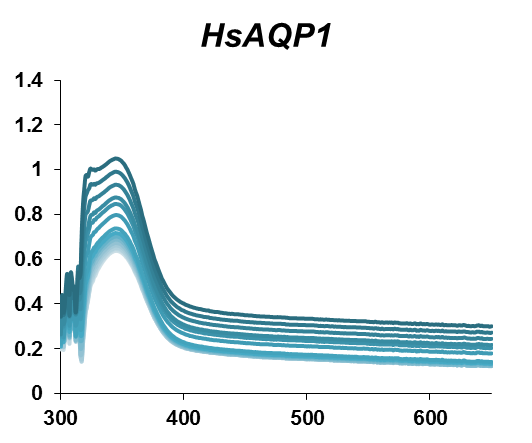

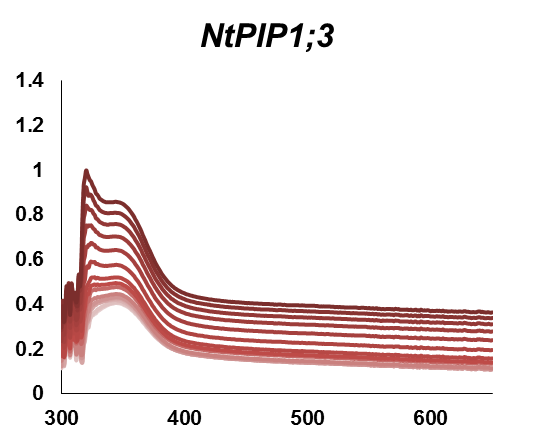

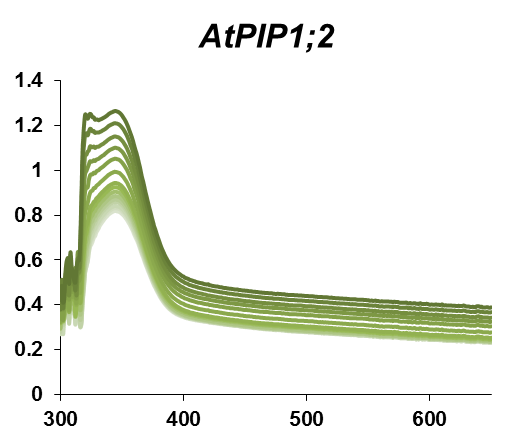


**A**

**B**

**C**

**D**

**E**

## Fig. S4 Scanning of absorbance spectra of yeast protoplasts from 300 nm to 650 nm during 10 min with multiple times of aeration.

(**A**) INVSc1, (**B**) Mock, (**C**) HsAQP1, (**D**) NtPIP1;3, (**E**) AtPIP1;2.


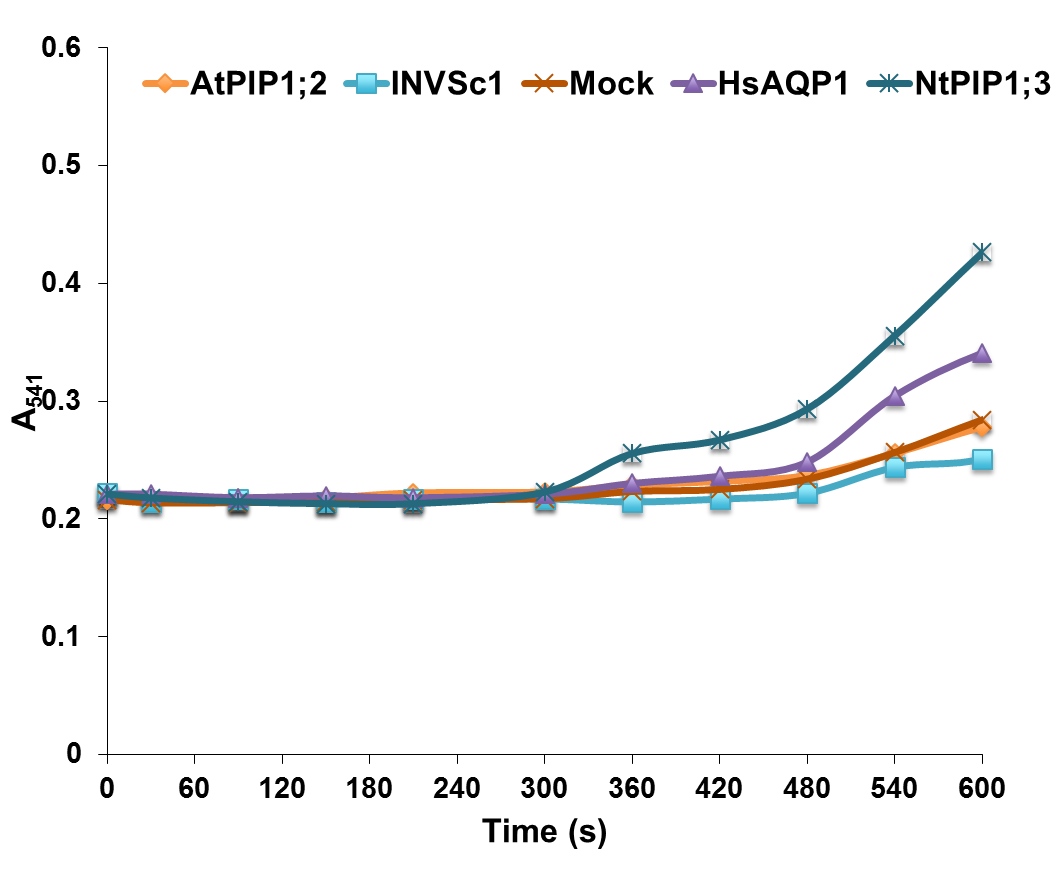

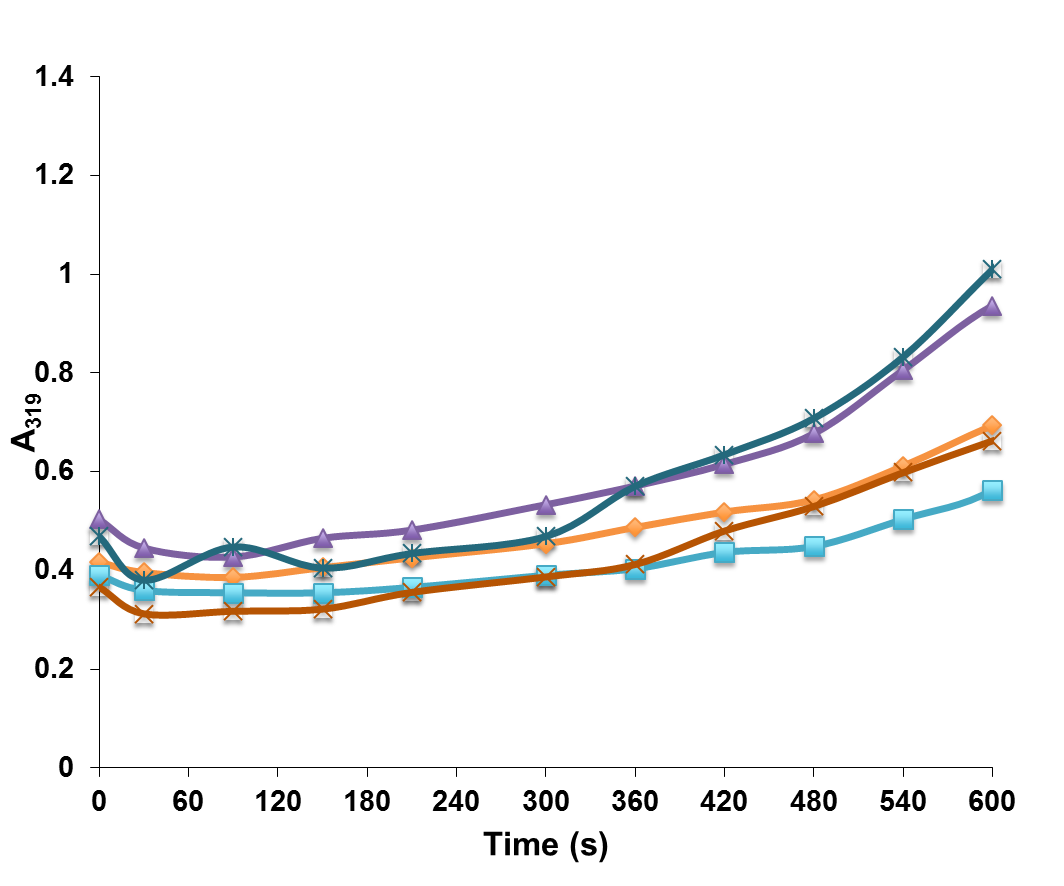

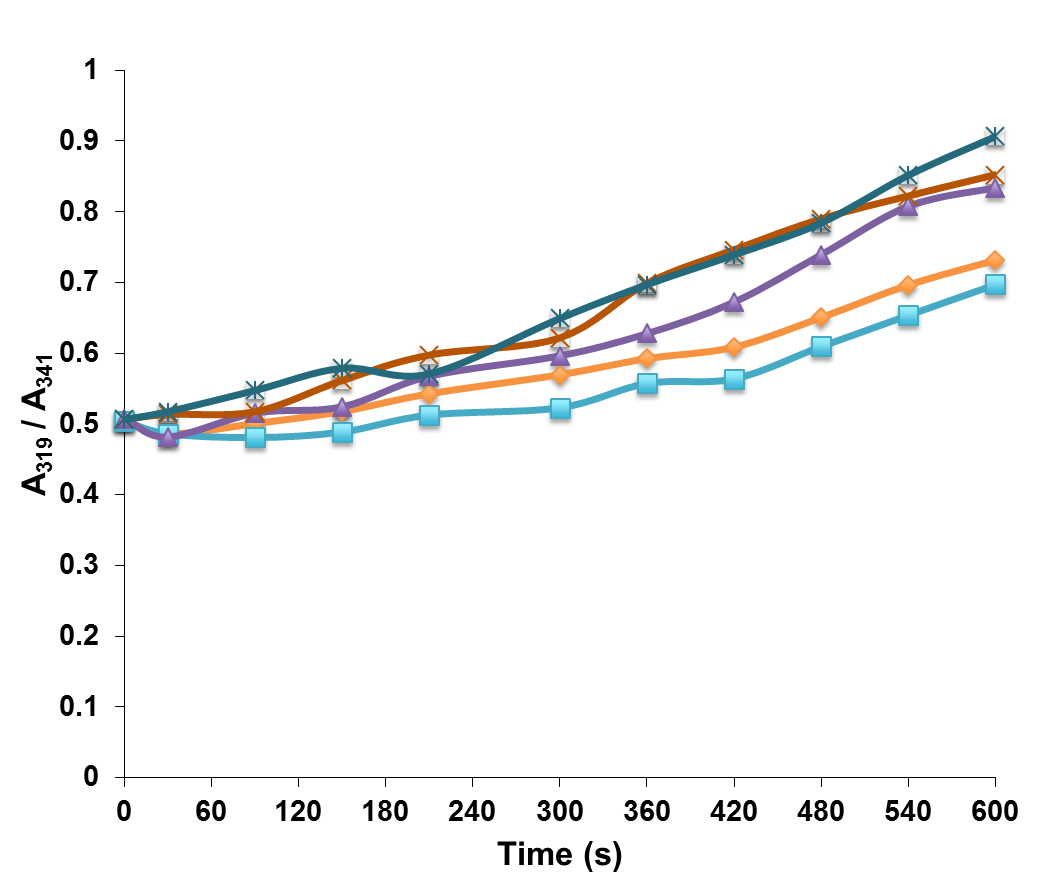


**A**

**B**

**C**

## Fig. S5 Change in A_541_, A_319_ and A_319_/A_341_ of yeast protoplasts during 10 min with multiple times of aeration.

(**A**) A_541_, (**B**) A_319_, (**C**) A_319_/A_341_.


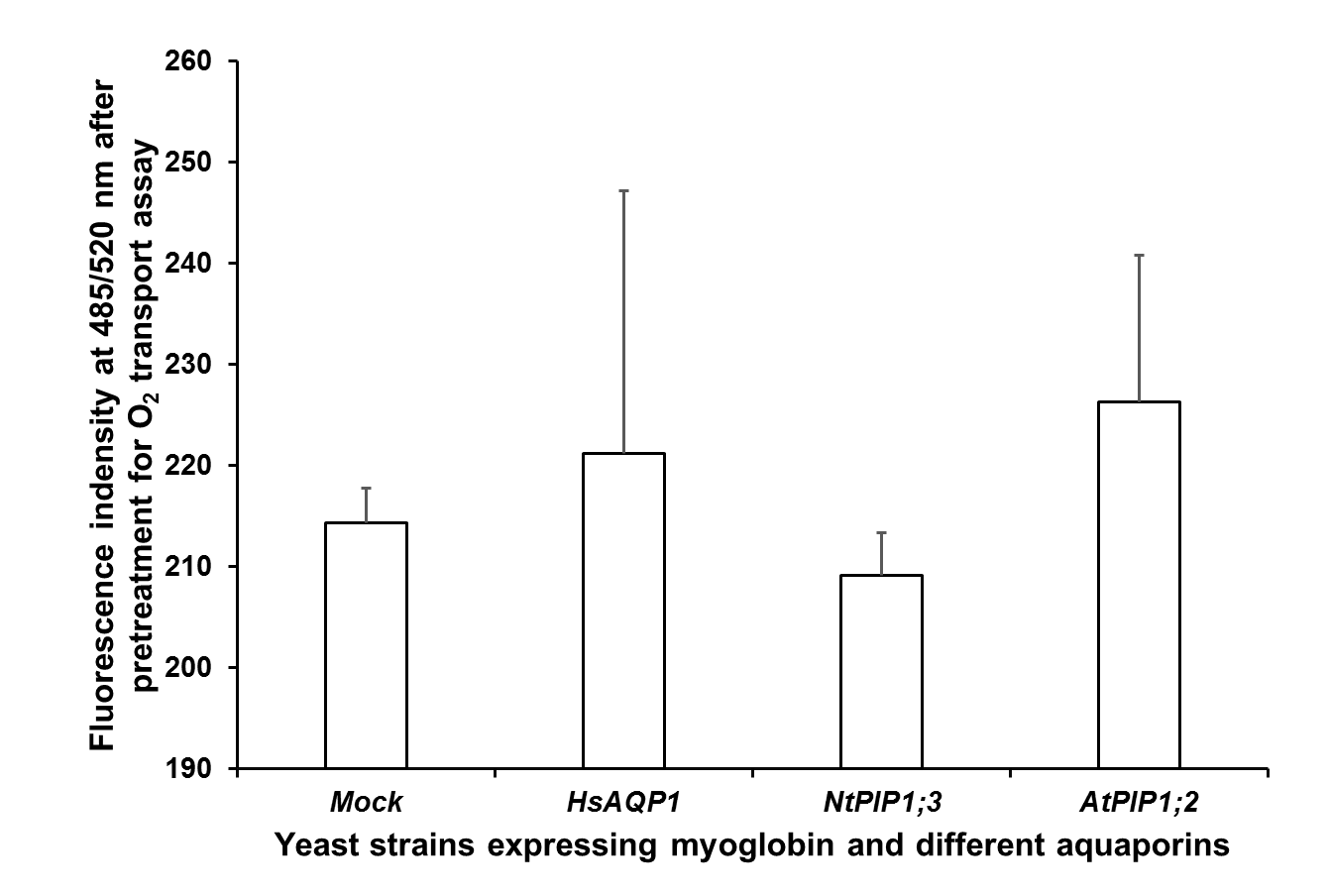


## Fig. S6 Cellular oxidative stress in yeast strains after pre-treatment for O_2_ transport assay using CM-H_2_DCFDA as the ROS indicator.

The bars show mean fluorescence intensity over 3 min (*n* = 6 ± SE).

## Fig. S7 Effect of the expression of NtPIP1;2 (NtAQP1), NtPIP1;3, HsAQP1, AtPIP1;2, and the mock control of empty expression vector on H_2_O_2_ permeability of yeast plasma membrane.

The bars show mean increases in fluorescence generated by CM-H_2_DCFDA caused by the influx of H_2_O_2_ (*n* = 6 ± SE). Different uppercase letters above bars indicate statistically significant differences between yeast strains under the same treatment at *P* ≤ 0.05, whereas different lowercase letters indicate statistically significant differences between treatments within each strain at *P* ≤ 0.05, as determined by ANOVA, Tukey’s test.


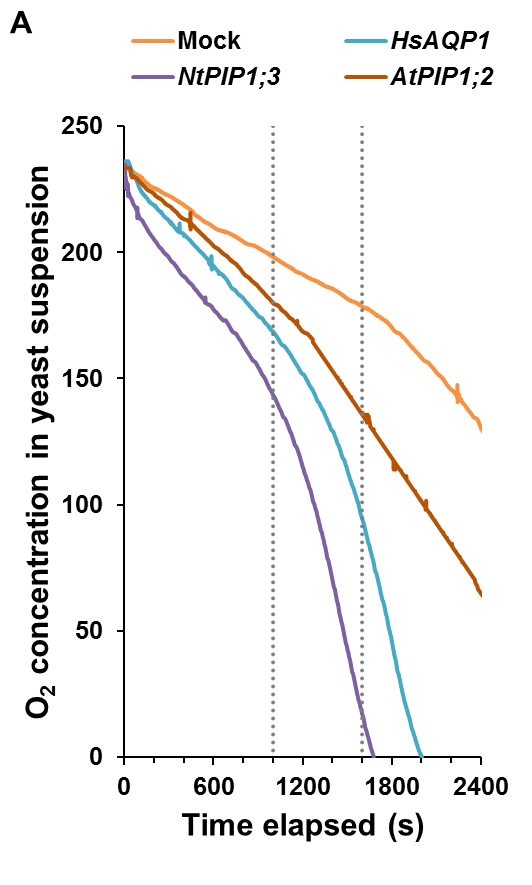

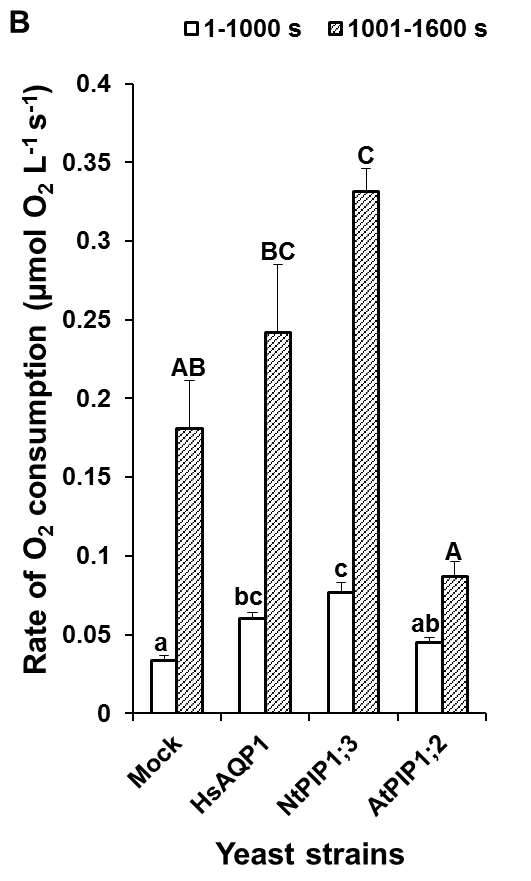


## Fig. S8 Characteristics of O_2_ consumption of yeast strains.

**(A)** Changes in concentration of soluble O_2_ over 40 min in cell suspension of yeast mock strain and the strains that expressed HsAQP1, NtPIP1;3 and AtPIP1;2 aquaporins, and **(B)** their rates of O_2_ consumption over 1600 s. Different lowercase letters above bars indicate statistically significant differences between yeast strains in the first 1000 s, whereas different uppercase letters indicate statistically significant differences between yeast strains in 1001-1600 s, as determined by ANOVA, Tukey’s test (*P* ≤ 0.05; *n* = 6 ± SE).

# Supplementary Tables

## Table S1 Subcellular localization of expressed aquaporin proteins predicted by TargetP 1.1 server

| Aquaporins | Length | mTP | SP | Other | Loc | RC |
| --- | --- | --- | --- | --- | --- | --- |
| HsAQP1 [*DQ895575*] | 269 | 0.016 | 0.927 | 0.171 | S | 2 |
| NtPIP1;3 [*U62280*] | 284 | 0.077 | 0.298 | 0.828 | - | 3 |
| AtPIP1;2 [*AT2G45960*] | 301 | 0.137 | 0.283 | 0.744 | - | 3 |

Note: Subcellular localization of the aquaporins was predicted using TargetP1.1. The highest score is the most likely location according to TargetP. Loc stands for the prediction of localization: M for mitochondrion, i.e. the sequence contains mTP, a mitochondrial targeting peptide; S for secretory pathway, i.e. the sequence contains SP, a signal peptide; - for any other location. RC stands for reliability class, from 1 to 5, where 1 indicates the strongest prediction.

## Table S2 The qPCR primers for gene transcript abundance assays in *Saccharomyces cerevisiae* and *Nicotiana* *tabacum*

|  | **Genes** | **Primers** | |
| --- | --- | --- | --- |
| *Saccharomyces cerevisiae* | *Myoglobin* | Forward | TCATCCATGTTCTGCATTCT |
|  |  | Reverse | GCTCGAGAGCTTTGTTCATA |
|  | *HsAQP1* | Forward | CTACACTGGCTGTGGGATTAACC |
|  |  | Reverse | GGTTGCTGAAGTTGTGTGTGATC |
|  | *NtPIP1;3* | Forward | TGGTATGATCTTTGCCCTTGTCT |
|  |  | Reverse | CAAATGTCACTGCTGGGTTAATG |
|  | *AtPIP1;2* | Forward | GTCCTCTTCTTTGCCTAATGGAGAC |
|  |  | Reverse | GTTGCCTGCTTGAGATAAACCCAG |
|  | *ScFBA1* | Forward | GCACCCAATCTCTCCAAACTTC |
|  |  | Reverse | TCTGGTCTCAAAGCGATGTCA |
| *Nicotiana tabacum* | *NtPIP1;1* | Forward | CCAATTACTGGAACTGGCATCA |
|  |  | Reverse | CCATGCCTGTTTTTTGTTGAAG |
|  | *NtPIP1;2* | Forward | TGCTGGTGTTGTGAAGGGATT |
|  |  | Reverse | AACATTAGCACCACCACCAAGTC |
|  | *NtPIP1;3* | Forward | TGGTATGATCTTTGCCCTTGTCT |
|  |  | Reverse | CAAATGTCACTGCTGGGTTAATG |
|  | *NtPIP1;4* | Forward | GCTGGTGTTGTGAAGGGATTC |
|  |  | Reverse | AACATTAGCACCACCACCAAGTC |
|  | *NtPIP2;1* | Forward | TTGGGTGCTGAGATTATTGGAA |
|  |  | Reverse | ATGGGAGTCACGGGCACTT |
|  | *EF1-α* | Forward | TGAGATGCACCACGAAGCTC |
|  |  | Reverse | CCAACATTGTCACCAGGAAGTG |
|  | *L25* | Forward | CCCCTCACCACAGAGTCTGC |
|  |  | Reverse | AAGGGTGTTGTTGTCCTCAATCTT |

# Supplementary Notes

## Note S1 Accession numbers and full-length open reading frames of the 20 aquaporin genes in this study

>HsAQP1[DQ895575][Homo sapiens]

ATGGCCAGCGAGTTCAAGAAGAAGCTCTTCTGGAGGGCAGTGGTGGCCGAGTTCCTGGCCACGACCCTCTTTGTCTTCATCAGCATCGGTTCTGCCCTGGGCTTCAAATACCCGGTGGGGAACAACCAGACGACGGTCCAGGACAACGTGAAGGTGTCGCTGGCCTTCGGGCTGAGCATCGCCACGCTGGCGCAGAGTGTGGGCCACATCAGCGGCGCCCACCTCAACCCGGCTGTCACACTGGGGCTGCTGCTCAGCTGCCAGATCAGCATCTTCCGTGCCCTCATGTACATCATCGCCCAGTGCGTGGGGGCCATCGTCGCCACCGCCATCCTCTCAGGCATCACCTCCTCCCTGACTGGGAACTCGCTTGGCCGCAATGACCTGGCTGATGGTGTGAACTCGGGCCAGGGCCTGGGCATCGAGATCATCGGGACCCTCCAGCTGGTGCTATGCGTGCTGGCTACTACCGACCGGAGGCGCCGTGACCTTGGTGGCTCAGCCCCCCTTGCCATCGGCCTCTCTGTAGCCCTTGGACACCTCCTGGCTATTGACTACACTGGCTGTGGGATTAACCCTGCTCGGTCCTTTGGCTCCGCGGTGATCACACACAACTTCAGCAACCACTGGATTTTCTGGGTGGGGCCATTCATCGGGGGAGCCCTGGCTGTACTCATCTACGACTTCATCCTGGCCCCACGCAGCAGTGACCTCACAGACCGCGTGAAGGTGTGGACCAGCGGCCAGGTGGAGGAGTATGACCTGGATGCCGACGACATCAACTCCAGGGTGGAGATGAAGCCCAAATAG

>HsAQP2 [CR542024][Homo sapiens]

ATGTGGGAGCTCCGCTCCATAGCCTTCTCCAGGGCTGTGTTCGCAGAGTTCCTGGCCACACTCCTCTTCGTCTTCTTTGGCCTCGGCTCTGCCCTCAACTGGCCACAGGCCCTGCCCTCTGTGCTACAGATTGCCATGGCGTTTGGCTTGGGTATTGGCACCCTGGTACAGGCTCTGGGCCACATAAGCGGGGCCCACATCAACCCTGCCGTGACTGTGGCCTGCCTGGTGGGCTGCCACGTCTCCGTTCTCCGAGCCGCCTTCTACGTGGCTGCCCAGCTGCTGGGGGCTGTGGCCGGAGCCGCTCTGCTCCATGAGATCACGCCAGCAGACATCCGCGGGGACCTGGCTGTCAATGCTCTCAGCAACAGCACGACGGCTGGCCAGGCGGTGACTGTGGAGCTCTTCCTGACACTGCAGCTGGTGCTCTGCATCTTCGCCTCCACCGATGAGCGCCGCGGAGAGAACCCGGGCACCCCTGCTCTCTCCATAGGCTTCTCTGTGGCCCTGGGCCACCTCCTTGGGATCCATTACACCGGCTGCTCTATGAATCCTGCCCGCTCCCTGGCTCCAGCTGTCGTCACTGGCAAATTTGATGACCACTGGGTCTTCTGGATCGGACCCCTGGTGGGCGCCATCCTGGGCTCCCTCCTCTACAACTACGTGCTGTTTCCGCCAGCCAAGAGCCTGTCGGAGCGCCTGGCAGTGCTGAAGGGCCTGGAGCCGGACACCGATTGGGAGGAGCGCGAGGTGCGACGGCGGCAGTCGGTGGAGCTGCACTCGCCGCAGAGCCTGCCACGGGGTACCAAGGCCTGA

>HsAQP3[CR541991][Homo sapiens]

ATGGGTCGACAGAAGGAGCTGGTGTCCCGCTGCGGGGAGATGCTCCACATCCGCTACCGGCTGCTCCGACAGGCGCTGGCCGAGTGCCTGGGGACCCTCATCCTGGTGATGTTTGGCTGTGGCTCCGTGGCCCAGGTTGTGCTCAGCCGGGGCACCCACGGTGGTTTCCTCACCATCAACCTGGCCTTTGGCTTTGCTGTCACTCTGGGCATCCTCATCGCTGGCCAGGTCTCTGGGGCCCACCTGAACCCTGCCGTGACCTTTGCCATGTGCTTCCTGGCTCGTGAGCCCTGGATCAAGCTGCCCATCTACACCCTGGCACAGACGCTGGGAGCCTTCTTGGGTGCTGGAATAGTTTTTGGGCTGTATTATGATGCAATCTGGCACTTCGCCGACAACCAGCTTTTTGTTTCGGGCCCCAATGGCACAGCCGGCATCTTTGCTACCTACCCCTCTGGACACTTGGATATGATCAATGGCTTCTTTGACCAGTTCATAGGCACAGCCTCCCTTATCGTGTGTGTGCTGGCCATTGTTGACCCCTACAACAACCCCGTCCCCCGAGGCCTGGAGGCCTTCACCGTGGGCCTGGTGGTCCTGGTCATTGGCACCTCCATGGGCTTCAACTCCGGCTATGCCGTCAACCCTGCCCGGGACTTTGGCCCCCGCCTTTTTACAGCCCTTGCGGGCTGGGGCTCTGCAGTCTTCACGACCGGCCAGCATTGGTGGTGGGTGCCCATCGTGTCCCCACTCCTGGGCTCCATTGCGGGTGTCTTCGTGTACCAGCTGATGATCGGCTGCCACCTGGAGCAGCCCCCACCCTCCAACGAGGAAGAGAATGTGAAGCTGGCCCATGTGAAGCACAAGGAGCAGATCTGA

>NtPIP1;1[AF440271][Nicotiana tabacum]

ATGGCAGAAAACAAAGAAGAAGATGTGAATCTTGGAGCAAACAAATACAGAGAAACACAACCCTTAGGAACAGCAGCACAAACAGAAAATAAGGATTATATTGAACCACCACCAGCACCATTATTTGAACCTGGTGAATTATCATCTTGGTCATTTTACAGAGCTGGGATTGCAGAATTTATGGCCACTTTCTTGTTCTTGTACATTACAATCTTGACTGTAATGGGACTTAAAAGGTCAGATAGTTTGTGTTCTTCTGTTGGTATTCAAGGTGTTGCTTGGGCTTTTGGTGGTATGATCTTTGCTCTTGTTTACTGTACTGCTGGTATCTCAGGAGGCCACATTAATCCAGCTGTGACCTTTGGTCTATTCTTAGCAAGGAAACTTTCCTTAACCAGGGCAGTATTCTACATGGTAATGCAATGCCTTGGTGCTATTTGTGGTGCTGGTGTTGTTAAAGGTTTCATGAAAGGTCCATACCAAAGACTTGGTGGTGGTGCCAATGTGGTTAACCCTGGCTATACTAAAGGTGATGGACTTGGTGCTGAAATTATTGGTACTTTTGTTCTTGTTTACACTGTTTTCTCTGCTACTGATGCCAAGAGAAATGCCAGAGATTCACATGTTCCTATTTTGGCACCTCTTCCTATTGGATTTGCTGTGTTCTTGGTTCATTTGGCCACCATCCCAATTACTGGAACTGGCATCAACCCTGCTAGGAGTCTTGGAGCTGCTATTATCTTCAACAAAAAACAGGCATGGGATGACCATTGGATCTTCTGGGTTGGACCATTCATTGGAGCTGCTCTTGCTGCAGTTTACCACCAGATTATTATCAGAGCCATTCCATTCAAGAGCTAA

>NtPIP1;2[AF024511][Nicotiana tabacum]

ATGGCAGAAAACAAAGAAGAAGATGTTAAGCTTGGAGCTAACAAATTCAGAGAAACACAGCCATTAGGAACAGCTGCTCAAACAGACAAAGATTACAAAGAACCACCACCAGCTCCTTTGTTTGAACCAGGGGAATTATCATCATGGTCATTTTACAGAGCTGGAATTGCAGAATTTATGGCTACTTTCTTGTTTTTGTACATCACTATCTTGACTGTTATGGGTCTTAAGAGATCTGATAGTCTGTGTAGTTCAGTTGGTATTCAAGGTGTTGCTTGGGCTTTTGGTGGTATGATCTTTGCTTTGGTTTACTGTACTGCTGGTATCTCAGGAGGACACATCAACCCAGCTGTGACCTTTGGATTGTTCTTGGCAAGGAAACTGTCCTTAACCAGGGCTATTTTCTACATAGTGATGCAATGCCTTGGTGCAATTTGTGGTGCTGGTGTTGTGAAGGGATTCATGGTTGGTCCATACCAGAGACTTGGTGGTGGTGCTAATGTTGTTAACCATGGTTACACCAAAGGTGATGGCCTTGGTGCTGAAATTATTGGCACTTTTGTCCTTGTTTACACTGTTTTCTCTGCTACTGATGCTAAGAGAAATGCCAGAGACTCATATGTTCCTATTTTGGCACCACTTCCCATCGGATTCGCGGTTTTCTTGGTTCATTTGGCCACCATTCCCATCACCGGAACTGGCATCAACCCCGCTAGGAGTCTTGGAGCTGCGATCATCTACAACACAGACCAGGCATGGGACGACCACTGGATCTTTTGGGTTGGACCATTCATTGGAGCTGCACTTGCTGCAGTTTACCATCAAATAATCATCAGAGCCATTCCATTCCACAAGTCGTCTTAA

>NtPIP1;3[U62280][Nicotiana tabacum]

ATGGCAGAGAACAAGGAAGAGGATGTTAAGCTAGGAGCAAACAAGTACAGAGAAACACAACCTTTGGGTACAGCAGCTCAAACAGACAAGGATTATAAGGAGCCACCACCAGCTCCTTTGGTTTGGAGGCAGAAGAGTTGTCGTCATGGTCTTTTTACAGAGCTGGAATTCAGCATGGCCACTTTCTTGTTCTTGTACATCACTATCTTGACTGTTATGGGTCTTAAAAGATCTGATAGTTTGTGTTCTTCTGTTGGTATTCAAGGAGTTGCTTGGGCTTTTGGTGGTATGATCTTTGCCCTTGTCTACTGCACTGCTGGTATCTCAGGAGGACACATTAACCCAGCAGTGACATTTGGTCTGTTCTTGGCAAGAAAGTTGTCTTTAACAAGGGCTGTGTTCTACATGGTGATGCAGTGCCTAGGTGCAATCTGTGGTGCTGGTGTTGTTAAAGGTTTTATGGTGGGTCCATACCAGAGACTTGGTGGTGGGGCCAACGTGGTTAACCCTGGCTACACTAAAGGTGATGGACTTGGTGCTGAGATTATTGGCACCTTTGTCCTTGTTTACACTGTTTTCTCTGCCACTGATGCCAAGAGAAATGCTAGAGATTCACATGTTCCTATTTTGGCACCTCTTCCTATTGGATTCGCGGTGTTCTTGGTTCATTTGGCCACCATCCCAATCACCGGAACCGGTATCACCCCCGCCCGGAGCCTTGGAGCTGCTATCATCTTCAACCAAGACCGGGCATGGGATGATCACTGGATCTTCTGGGTTGGACCATTCATTGGAGCTGCACTTGCTGCAGTTTACCACCAGATAATCATCAGAGCCATTGCATTCAAGAGCTAA

>NtPIP1;4[DQ914525][Nicotiana tabacum]

ATGGCAGAAAACAAAGAAGAAGATGTTAAGCTTGGAGCTAACAAATTCAGAGAAACACAGCCATTAGGAACAGCTGCTCAAACAGACAAAGATTACAAAGAACCACCACCAGCTCCTTTGTTTGAACCAGGGGAATTATCATCATGGTCATTTTACAGAGCTGGAATTGCAGAATTTATGGCTACTTTCTTGTTTTTGTACATCACTATCTTGACTGTTATGGGTCTTAAGAGATCTGATAGTCTGTGTAGTTCAGTTGGTATTCAAGGTGTTGCTTGGGCTTTTGGTGGTATGATCTTTGCTTTGGTTTACTGTACTGCTGGTATCTCAGGAGGACACATCAACCCAGCTGTGACCTTTGGATTGTTCTTGGCAAGGAAACTGTCCTTAACCAGGGCTATTTTCTACATAGTGATGCAATGCCTTGGTGCAATTTGTGGTGCTGGTGTTGTGAAGGGATTCATGGTTGGTCCATACCAGAGACTTGGTGGTGGTGCTAATGTTGTTAACCATGGTTACACCAAAGGTGATGGCCTTGGTGCTGAAATTATTGGCACTTTTGTCCTTGTTTACACTGTTTTCTCTGCTACTGATGCTAAGAGAAATGCCAGAGACTCACATGTTCCTATTTTGGCACCACTTCCCATCGGATTCGCGGTTTTCTTGGTTCATTTGGCCACCATTCCCATCACCGGAACTGGCATCAACCCCGCTAGGAGTCTTGGAGCTGCGATCATCTACAACACAGACCAGGCATGGGACGACCACTGGATCTTTTGGGTTGGACCATTCATTGGAGCTGCACTTGCTGCAGTTTACCATCAAATAATCATCAGAGCCATTCCATTCCACAAGTCTTCTTAA

>NtPIP2;1[AF440272][Nicotiana tabacum]

ATGTCAAAGGACGTGATTGAAGAAGGACAAGTTCATCAACAGCACGGGAAGGATTACGTGGACCCACCACCAGCTCCTTTGCTTGATTTTGCAGAACTCAAGCTCTGGTCTTTTCACAGAGCTCTTATTGCTGAGTTCATTGCTACTCTTCTTTTCCTTTACGTCACTGTTGCAACTGTAATTGGTCACAAGAAGTTGAATGGTGCTGATAAATGTGATGGGGTTGGTATTCTTGGTATTTCTTGGGCTTTTGGTGGCATGATTTTTGTTCTTGTTTACTGCACTGCCGGTATCTCTGGTGGACACATTAACCCAGCAGTGACATTTGGGTTGTTCTTAGCAAGAAAAGTGTCATTGTTAAGAGCAGTGGGATATATTATTGCACAGTCATTAGGTGCAATTTGTGGTGTTGGTTTAGTGAAAGGTTTCATGAAACATTACTACAACACATTAGGTGGTGGTGCTAATTTTGTGCAACCTGGTTATAACAAGGGCACAGCTTTGGGTGCTGAGATTATTGGAACTTTTGTTCTTGTTTACACTGTTTTCTCTGCTACTGACCCTAAAAGAAGTGCCCGTGACTCCCATGTCCCTGTTTTGGCCCCTCTGCCAATTGGTTTTGCTGTTTTCATGGTTCATTTGGCTACTATTCCTATTACTGGAACTGGTATTAACCCTGCTAGGACCTTTGGAGCTGCTGTCATTTACAACACTGAAAAAATCTGGGATGATCAATGGATTTTCTGGGTTGGACCATTTGTGGGAGCATTGGTAGCAGCAGTATATCATCAGTATATCTTGAGAGGTTCAGCAATTAAGGCATTGGGTTCTTTCCGCAGTAACCCAACCAACTAA

>NtXIP1;1[HM475294][Nicotiana tabacum]

ATGGCTTCCAATGCTAGTCATGTTTTAGGCGATGAAGAAAGCCAACTTTCTGGTGGAAGTAATAGAGTTCAACCTTTCTCTTCTACACCAAAAAACAGGAATATTGATGATGAGGGAAAGAAGCATACTTCTCTCACAGTGGCACAAAGGCTGGGCATTTCTGACTTCTTTTCTTTGGATGTATGGCGAGCGTCAGTGGGAGAGCTCCTAGGCTCGGCGGTTCTTGTTTTTATGTTGGACACCATAGTGATCTCCACCTTTGAAAGTGATGTGAAAATGCCAAATTTGATCATGTCAATTCTCATAGCAATTGTGATCACAATTCTACTCCTCGCCGTTGTTCCGGTGTCCGGTGGCCACATAAACCCCGTCATCTCCTTCTCCGCCGCGCTTGTCGGAATTATATCCATGTCAAGAGCCATTATTTACATGGTGGCACAATGTGTTGGAGCAATTTTAGGTGCACTAGCTCTAAAAGCAGTAGTTAGCTCTACTATTGCACAAACTTTCTCACTTGGTGGTTGTACCATAACAGTAATTGCACCGGGCCCAAATGGGCCCATTACAGTGGGCCTAGAAATGGCCCAAGCTTTGTGGCTTGAGATCTTTTGTACATTTGTTTTTCTTTTTGCTTCAATTTGGATGGCTTATGATCATAGGCAAGCTAAGGCCCTTGGCCTTGTCACTGTCTTGTCCATTGTTGGTATAGTTTTGGGCCTTCTTGTGTTCATCTCGACTACGGTCACCATGAAAAAGGGCTACGCCGGAGCGGGGATGAATCCGGCGAGGTGTTTCGGGGCTGCTGTTGTTAGAGGAGGTCATCTTTGGGATGGGCATTGGATCTTTTGGGTTGGGCCTACTATTGCTTGTGTAGCATTTTATGTGTACACAAAGATAATTCCACCAAAGCATTTTCATGCAGATGGATACAAGTATGATTTTATTGGAGTTGTTAAGGCTTCGTTTGGGTTGCATGAATGA

>AtPIP1;1[AT3G61430][Arabidopsis thaliana]

ATGGAAGGCAAGGAAGAAGACGTTAGAGTTGGAGCTAACAAGTTCCCGGAGAGACAACCAATCGGAACATCAGCTCAGAGTGACAAGGACTACAAGGAACCACCACCAGCTCCGTTTTTCGAACCTGGTGAGCTTTCTTCATGGTCTTTTTGGAGAGCTGGGATCGCTGAGTTCATCGCTACTTTTCTCTTTCTCTACATCACTGTCTTGACTGTTATGGGAGTGAAAAGGTCACCGAACATGTGTGCTTCCGTCGGAATCCAAGGAATCGCTTGGGCTTTCGGTGGTATGATATTTGCCTTAGTCTACTGTACCGCTGGTATCTCCGGTGGACACATCAACCCAGCGGTTACTTTTGGTCTGTTCTTAGCCCGGAAGCTGTCGCTTACTAGAGCTCTGTACTACATAGTGATGCAGTGCTTGGGAGCTATCTGTGGTGCTGGAGTGGTTAAAGGGTTCCAGCCTAAGCAATACCAGGCTCTAGGAGGAGGAGCTAACACTGTGGCTCATGGTTACACCAAGGGAAGTGGTCTTGGAGCTGAGATCATTGGCACATTCGTTCTTGTATACACAGTCTTCTCAGCAACTGACGCCAAGAGAAATGCTCGTGACTCTCATGTCCCTATTCTTGCACCACTCCCAATCGGGTTTGCGGTTTTCTTGGTTCACTTGGCAACCATCCCAATCACTGGCACAGGCATCAACCCAGCTAGAAGCCTTGGAGCTGCAATCATCTACAACAAAGACCATTCCTGGGATGACCACTGGGTGTTTTGGGTTGGCCCCTTCATTGGAGCTGCACTTGCTGCTCTTTACCATGTGGTTGTCATCAGAGCCATCCCCTTCAAGTCCAGAAGCTAA

>AtPIP1;2[AT2G45960][Arabidopsis thaliana]

ATGGAAGGTAAAGAAGAAGATGTTAGAGTCGGAGCTAACAAGTTTCCGGAGAGGCAACCGATCGGAACTTCGGCTCAGAGTGACAAGGACTACAAAGAGCCACCACCTGCGCCGTTGTTCGAGCCCGGCGAGCTAGCTTCATGGTCCTTCTGGAGAGCTGGGATTGCTGAGTTTATAGCTACGTTTTTGTTCCTGTACATCACTGTTTTGACTGTTATGGGTGTGAAGAGGTCACCGAACATGTGTGCTTCCGTCGGAATCCAAGGTATCGCTTGGGCTTTCGGTGGTATGATCTTCGCTCTCGTCTACTGCACCGCTGGTATCTCCGGTGGACACATCAACCCAGCGGTTACGTTCGGTTTGTTCTTAGCTAGGAAGCTTTCGCTCACACGAGCTGTGTACTACATAGTGATGCAGTGCTTAGGAGCTATCTGTGGAGCTGGTGTGGTCAAGGGGTTCCAGCCAAAGCAATACCAGGCTTTGGGAGGTGGAGCCAACACCATAGCTCATGGCTACACCAAAGGAAGTGGTCTTGGAGCTGAGATTATTGGAACCTTTGTCCTTGTTTACACCGTCTTCTCTGCCACTGATGCCAAGAGAAACGCTCGTGACTCTCATGTTCCTATTCTAGCACCGCTCCCTATCGGATTCGCTGTGTTCTTGGTTCACTTAGCAACCATCCCCATTACTGGAACTGGAATCAACCCAGCAAGAAGTCTTGGAGCTGCAATCATCTTCAACAAGGACAACGCTTGGGATGACCACGTGATGGGTCTTTTGGGTTGGACCATTCATTGGTGCTGCACTTGCTGCTCTCTACCACGTTATAGTCATCAGAGCCATCCCATTCAAGTCCAGAAGCTAAAGCTGATTGAGTTCTATTTAAAATCTGGCTTTTGTTCTTAG

>AtPIP1;3[AT1G01620][Arabidopsis thaliana]

ATGGAAGGGAAAGAAGAGGATGTTCGAGTGGGAGCTAACAAGTTCCCGGAGAGGCAACCGATAGGTACATCGGCTCAGACGGACAAAGACTACAAGGAGCCACCACCAGCTCCATTTTTCGAGCCAGGCGAGCTGAGTTCGTGGTCCTTCTACAGAGCCGGAATCGCCGAGTTCATAGCCACCTTCCTGTTTCTATACATAACAGTATTGACAGTGATGGGAGTGAAGAGAGCACCAAACATGTGTGCCTCTGTTGGAATCCAAGGCATTGCTTGGGCTTTCGGTGGCATGATCTTTGCCCTTGTCTACTGTACTGCTGGAATCTCTGGTGGGCACATAAACCCAGCGGTGACATTTGGTCTGTTCTTGGCTCGTAAGCTGTCATTGACGAGAGCTGTCTTTTACATCGTGATGCAATGTCTCGGAGCCATCTGCGGCGCCGGAGTTGTCAAAGGCTTCCAGCCAAATCCTTACCAAACTCTCGGCGGAGGAGCCAACACAGTCGCTCACGGCTACACTAAGGGCTCTGGTTTGGGTGCTGAGATAATCGGAACCTTCGTCCTTGTCTACACGGTCTTCTCCGCCACTGACGCCAAGAGAAGCGCTCGTGACTCCCACGTTCCGATTTTGGCACCACTCCCAATCGGATTCGCTGTGTTCTTGGTTCACTTGGCGACGATTCCAATCACCGGAACAGGAATTAACCCAGCTAGGAGTCTTGGAGCTGCAATCATCTACAACAAGGACCACGCTTGGGACGACCACTGGATATTCTGGGTCGGACCATTCATTGGAGCAGCTCTTGCGGCTCTTTACCACCAACTTGTCATCAGAGCCATTCCATTCAAGTCCAGATCCTGA

>AtPIP1;4[AT4G00430][Arabidopsis thaliana]

ATGGAAGGCAAAGAAGAAGATGTACGAGTGGGAGCTAACAAGTTCCCGGAGAGGCAACCCATCGGTACATCGGCTCAGTCCACCGACAAGGACTACAAAGAGCCACCTCCTGCGCCACTGTTCGAGCCCGGCGAGCTCAGCTCATGGTCTTTCTACAGAGCCGGAATAGCTGAGTTTATCGCTACTTTCTTGTTTCTCTACATCACTGTTTTGACTGTAATGGGAGTTAAGAGAGCACCAAACATGTGTGCTTCTGTTGGAATCCAAGGTATCGCTTGGGCTTTTGGTGGCATGATCTTTGCTCTTGTCTACTGTACTGCTGGAATTTCAGGTGGACACATCAACCCTGCTGTAACATTCGGTCTGTTCTTGGCTCGGAAGTTATCTCTGACCAGAGCAGTGTTCTACATGATTATGCAATGTCTTGGAGCCATCTGTGGTGCCGGAGTCGTCAAAGGTTTCCAGCCAACGCCGTACCAGACTCTCGGTGGTGGTGCTAACACCGTTGCTCATGGCTACACCAAAGGTTCTGGCCTTGGTGCTGAAATCATCGGAACATTCGTTCTCGTCTACACTGTCTTCTCCGCCACCGACGCCAAGAGAAGCGCCCGTGACTCACACGTCCCGATTTTGGCGCCGCTCCCAATCGGATTTGCAGTGTTCTTGGTACACTTGGCAACAATACCGATCACCGGGACCGGAATCAACCCAGCTAGAAGTCTTGGAGCCGCAATTATCTACAACAAGGACCACTCTTGGGATGACCATTGGATTTTCTGGGTTGGACCATTCATTGGAGCAGCTCTAGCAGCACTATATCACCAGATTGTCATCAGAGCGATTCCTTTCAAGAGCAAGAGTTAG

>AtPIP1;5[AT4G23400][Arabidopsis thaliana]

ATGGAAGGCAAAGAAGAAGACGTCAATGTTGGAGCCAACAAGTTCCCAGAGAGACAGCCGATCGGTACGGCGGCTCAGACGGAGAGCAAGGACTATAAGGAACCACCACCGGCGCCGTTTTTCGAACCCGGCGAGCTCAAATCTTGGTCTTTCTACAGAGCAGGGATAGCTGAGTTCATAGCCACTTTCCTTTTCCTCTACGTCACCGTTTTGACAGTCATGGGTGTTAAGAGAGCTCCCAATATGTGTGCCTCTGTTGGAATCCAAGGCATCGCTTGGGCTTTTGGTGGCATGATCTTTGCTCTTGTTTACTGTACTGCTGGAATCTCAGGAGGACATATTAATCCGGCGGTGACTTTTGGTTTGTTCTTGGCGAGGAAGCTATCTTTAACCAGAGCTCTGTTCTACATAGTAATGCAGTGCCTTGGAGCTATATGTGGTGCTGGTGTGGTTAAAGGGTTTCAACCAGGGCTGTACCAGACGAATGGCGGTGGAGCTAATGTGGTGGCTCATGGTTACACAAAGGGTTCAGGTCTTGGTGCAGAGATTGTTGGAACTTTTGTTCTGGTTTACACTGTTTTCTCAGCTACTGATGCTAAGAGAAGTGCCAGAGACTCTCATGTCCCTATCTTGGCTCCGCTTCCAATTGGGTTTGCTGTCTTCTTGGTGCACTTGGCTACCATCCCAATTACTGGAACTGGCATTAACCCGGCCAGGAGTCTCGGAGCTGCCATCATCTACAACAAGGATCATGCTTGGGATGACCATTGGATCTTCTGGGTCGGTCCATTCATTGGTGCTGCGCTTGCTGCTCTGTACCATCAGATAGTCATCAGAGCTATTCCTTTCAAGTCCAAGACATAA

>AtNIP2;1[AT2G34390][Arabidopsis thaliana]

ATGGATGACATATCAGTGAGCAAAAGCAACCACGGAAATGTTGTTGTGCTCAACATCAAAGCGTCTTCTCTTGCAGACACTTCTCTTCCTTCCAACAAACACGAGTCATCGTCCCCGCCTTTGCTCTCTGTCCATTTCTTGCAAAAGCTCTTAGCGGAGCTCGTTGGAACTTACTATCTGATATTTGCTGGCTGTGCCGCCATTGCCGTGAATGCTCAACACAACCATGTAGTGACTCTTGTGGGGATTGCTGTGGTTTGGGGTATAGTGATAATGGTTCTTGTTTACTGTCTCGGCCACCTCTCTGCACATTTCAATCCTGCTGTCACTCTTGCCTTAGCATCTTCCCAAAGATTCCCTCTGAATCAAGTCCCGGCCTACATAACTGTTCAAGTCATCGGATCAACCTTGGCATCTGCGACTCTGCGTCTTCTGTTTGATCTGAACAATGATGTGTGCAGCAAGAAACATGATGTCTTCCTCGGGTCATCTCCTTCTGGGTCTGATCTTCAAGCATTCGTGATGGAGTTCATCATTACCGGCTTCCTCATGTTAGTCGTCTGTGCCGTCACAACCACCAAGAGAACTACAGAGGAACTAGAGGGGCTAATCATTGGGGCAACCGTCACACTGAATGTTATATTTGCTGGAGAGGTATCAGGAGCATCGATGAATCCAGCCAGAAGCATAGGACCTGCACTTGTGTGGGGATGTTACAAAGGGATTTGGATATACTTGCTGGCTCCAACACTTGGTGCCGTCTCTGGGGCCTTGATTCATAAAATGTTACCGTCAATACAGAACGCAGAGCCTGAGTTCTCCAAGACAGGATCTTCTCATAAACGAGTTACCGATCTTCCTCTGTGA

>LbAQP1[JQ585592][Laccaria bicolor]

ATGCATCCACAAGTTGCTTCACTCTTCGACAACGTCTACGAGGATCTGGCCGCAGCTACCCTAGAGTTCATTGGCACGGCGTTTTTCCTTTTGTTCGGTCTGGGGGGTATTCAGGCTAGCACCGCGGAGGACACGGCGAGCAGTCAGCCACCAGCCTCGGGCATCGAACATGTTCTCTATATCTCAACCTGCATGGGGTTCTCTCTCGTTGTATCCGCCTGGCTCTTCTTCCGCGTCACTGGTGGACTCTTCAATCCAAATATATCTTTTGCGTTGCTTCTAGTCGGGGGTCTCAAGCCACTTCGCTTCGTGCTGTTCTGCATTGCTCAATTGACTGGTGCGATCGCAGGAGCTGCCATCGTTCGCGGTCTGACGTCGGCGCCCCTCTCTGTCAACAACGTTCTTCAGCAAGGGACGAGTGCCGCACAAGGCGTTTTCATTGAGATGTTTATCACCGCGGCGCTTGTGCTTTCCGTTTTGATGTTAGCGGCAGAGAAACATGAGGCCACTCCTTTCGCTCCCGTCGGGATTGGTCTTACGCTCTTTGCTTGTCATCTTTTTGCGGTTTACTACACTGGCGCTGCTATGAATTCAGCAAGGGCGTTTGGACCAGCTGTAATCTCCGGATTCCCAGAGCCCCAACACTGGGTGTATTGGGTTGGGCCGTTCTTGGGATCACTCCTCGGTGCAGGCTTCTACGCTACCTTGAAGCACTACAAGTATTGGCGTCTCAATCCCGATCAAGCTACCAGTGATTACAGGAAATCGCCTTCAGATCCAGTGGCCCTGCTGAAATCAACTGCGGAAACCTTCATCAATGTCGGAGACGAAGAGACTCGCAATGGGTGTGCGTCAAATGAGGAAGGGGTCAGGGCGACGGGCGATGAAAAGTCGAGCAACGCGACCTCGTCACGCACGAATTTCAGCCCGGTCTAA

>LbAQP3[JQ585593][Laccaria bicolor]

ATGTCCGCTACTCCAATCATCCACCTGCGCGACGTGAAAAAGCGTACTGGAGTCTTGAACGCATGGGAGAGGGTACGGAACAAGCCCCAGGTGCACTGGGCGATGGAGTGTTTCGCTGAGGCTTTGGGCGTCTTTTTCTACGTATACTTTGGACTCGGATCTACCGCAGCTTGGGTGATTGGGAACATCTTGAAACAGTCTGGGCTCTCCTCTGTCTTCCAGATCGGTTTCGCCTACGCATTTGGCATTTTGTTTGCCATCGGTGTCTGTGCAGCTACTTCTGGTGGACACTTCAACCCTTGCGTTACCATCGCATTCACGATATTCAGAGGTTTTCCACCCCTGAAGGCTGTCAGATATATAGTTGCGCAAATTCTTGGAGCTTACATTGCGTCCGCCCTTGTATACAATCAATGGAAGGTCCTTATCGTGGAGTCGGAACTTCTCTTGAAACAAGCTGGCGTCTACGAAACGACGATGTTCACGCCCAATGGTCCGGCAGGAATCTTCGCTCTTTATCTTCTTCCTGGAGCGCAAACTTTGCCTCGCGCTTTCCTTAATGAATTCGTTAATTGTTTTGTGCTCGCCTTGGTTATCTGGGCTGCTCTTGACCCTACTAGTTTCATGATTCCACCCGTTATGGCTCCTTTCATCATCGCTGCGGCATACGCTGGCTCTATCTGGGGTTATGCGGTTCCCGCGATTTCTTTGAATTCGGCCCGTGACATTGGTTGCCGTTTGTTCGCACTGACCATCTGGGGAAAGTCAGCTGCGGGAGGATCCTACTCGGCAATAGCGGCACTTGTAAATATTCCAGCCACTTTGCTCGCTGCGGTCGTCTATGAGCTGTTCCTCGTGGATTCTGATCGAGTTGTAGCTGGCTCACATCTTGAGTTCATGAACGTTGCAGCAAATCACCGAAGGCACCGTCAGCAGGCCGAGGATGACAACCTTGTCGAAGCTGATGACTCATCGCAAGAGAAGCCTGTATGA

>LbAQP5[JQ585594][Laccaria bicolor]

ATGGCCGAATTTGTTGGTGTGGCACTCTTGGTTATCTTTGGCGCGGGGGCTGCTTGCCAGGTTATCCTCTCGACAAATCCAGGCGTCTCACCCTCCGAACGAGGTTCGTTTCTTTCGATCAATTTCGGATGGGCAATCGGTATCGCTACGGGTGCCTGGGTCAGCACTGGCATGTCTGAAGGACACATAAACCCTGCAATTACAATTGGGATGGCGACGTACCGCGGGTTTCCCTGGCGTGAAGTACCCGGCTATATCTTCGGCCAGGTGTTGGGTGGGTTTGTTGGTGCAGCACTGGTGTACGCAAATTATTTCCATGCAATTGATATCTTCGAAGGAGGGCACCGCACGCAAGCCACTGCTTCTCTCTTTGCGACATTTGCTCTGCCGTACATGACACAAGCATCATGTTTCTTTTCAGAGTTTTTAGCCACCGCCGTCCTTTTCATCGTGTTCTTGGCTCTCAACGATAAGCATAATGGCGCACTCACAAACGGGCTCCTACCATTTGCCCTGTTTATTTTGTTCATTGGTCTTGGGGCATCGCTCGGCATGCAAACAGGTTATGCCGTCAACCCAGCGAGAGACTTTGGACCGCGCTTGTTCCTTGCTATGGCAGGCTACGGAAAGGCCGTTTTCAACTATCGCAGACAATATTGGATTTGGGCACCCATAATTGCTCCAATCCTTGGCGCTCAAGCCGGAGGCCTGCTCTATGATACCTTTATATATAATGGAGATGACAGTCCAATCAAGTGGCGGTAG

>LbAQP6[JQ585595][Laccaria bicolor]

ATGGACGACAAATTCGACGACGACGCTCTCCCCAACTCAAAGACTACGGCTAAGGACTACGAGGACAAGCTCCCAGAATATGATTATACCACCACATTCCCCAATACGTGGATGAGACTACGTGAACCCTTTCGTGAATATTTCGCAGAGTTCGTTGGTGTTGCGGTCCTTATCATCTTTGGTGTCGGTGCCGACTGTCAAGTCGTCTTGTCTGCAAACACTGGCGTTGCATCATCTCCGAAAGGTAGCTATCTATCACTGAATTGCGGTTGGGCCATTGGCACAGCTATGGGCGTTTGGATCTCGGGCGGAATTTCAGGCGGTCATATTAACCCTGCTGTAACACTGGCGATGGCGACATGGCGCGGCTTCCCATGGTGGAAAGTTCCCGGTTTCATTTTCGCTCAGCTCCTAGGCGGAATAGTCGGAGCTGGACTGGTCTATGTGAATTACATTCACGCCATTGATATCGTAGAAGGCGGCCGCCATATCCGAACCCTCGATACCGCTGGATTGTTCGCAACGTATGCAGCTGATTACATGACGAACTTGTCTTGCTTTTTCTCAGAGTTCCTCGCTACTGCCGTGCTTATCATCGTCATCCACGCGATGAACGACAAGAGAAACACCCCTCCTCCAGCTGGCATCGTACCATTTGTTCTCTTCTTCCTTATCCTTGGTATCGGTGCATCCCTTGGAATGGAAACAGGTTACGCCATTAACCCCGCTCGTGATCTTGGCCCCCGCATGCTCACTGCTATGGTTGGCTATGGAAGACAGGTTTTCGCTTTCCGGAATCAATATTGGATCTGGTGTCCAGTTCTTGCCCCATTCCTGGGCGCTCAGGTTGGAACGATCTTCTATGACCTGTTCTTCTACAAAGGACAAGATAATGTTTTCGGGCGATTAGGGTCACACATACACATCTCCCCAGCTTAA

>LbAQP7[JQ585596][Laccaria bicolor]

ATGTCTGGCCAACATCAGATCACTGAGCAACCGTCTGGAAACCCACTCTCCAGAACTTCTACACTTATTCAAGAGAAACCGCTGACTCCCACATCGTCTCACGCTGAGACTCAAAAACATCTCGAGGCCCCTCGACAGTCTTCTTTTCTTATCCAACTGCAAGATATTAGGCATGCAATCCGCATGCCCATGGCCGAATTTTTCGGTGTGGCGCTCTTGATCATTTTCGGTGCAGGGTCTGCCTGCCAGGTTGTACTCTCGACAAATCCAAACGTCGCATCATCTGATCGAGGTTCATTTCTCTCCATAAACCTCGGATGGGCCATCGGTATTGCCATGGGTGCCTGGGTCAGCGGCGGCATCTCTGGAGGACACATTAACCCTGCGATAACAATCGCAATGGCGACTTATCGCGGCTTTCCTTGGCGTAGAGTGCCCAGCTACATCTTCGCCCAGGTGTTAGGTGGGGTCGTTGGTGCCGCGCTGGTATACGCGAATTATATCCATGCAATCGATATCTTTGAAGGCGGACGTCACGTCCGCACCCAAGCTACTGCTTCTCTCTTCGCAACGTACGCTCTGCCATACATGACACAAGTATCATGTTTCTTTTCGGAATTCTTGGCCACCGCCGTTCTTTCTATGATGGTTTTGGCCCTCACCGATAACCGCAATGGCGCTCCGACAAATGGGCTTTTACCATTTGCACTATTTGTTTTGTTCATCGGCCTTGGGGCGTCGCTCGGCATGGAAACAGCGTACGCCCTCAATCCTGCGCGAGACTTTGGACCACGCTTGTTCCTCGCTATGTCAGGTTACGGAAAGGCTCTCTTCAACTATCGCAGTCAATATTGGCTCTGGGCACCCATTATTGCTCCGGTCCTTGGCGCTCAGGCTGGAGGCTTACTTTATGACACCTTTTTATACGATGGAGATAACAGCCCCATCAAATGGCGCCGCGCTTCCTCGCAAGAATGCCAGCTCGCTGAGGTTGTTTGA

## Note S2 Amino acid sequences of aquaporins in this study deduced by NCBI ORF Finder

>AtPIP1;1

MEGKEEDVRVGANKFPERQPIGTSAQSDKDYKEPPPAPFFEPGELSSWSFWRAGIAEFIATFLFLYITVLTVMGVKRSPNMCASVGIQGIAWAFGGMIFALVYCTAGISGGHINPAVTFGLFLARKLSLTRALYYIVMQCLGAICGAGVVKGFQPKQYQALGGGANTVAHGYTKGSGLGAEIIGTFVLVYTVFSATDAKRNARDSHVPILAPLPIGFAVFLVHLATIPITGTGINPARSLGAAIIYNKDHSWDDHWVFWVGPFIGAALAALYHVVVIRAIPFKSRS*

>AtPIP1;2

MEGKEEDVRVGANKFPERQPIGTSAQSDKDYKEPPPAPLFEPGELASWSFWRAGIAEFIATFLFLYITVLTVMGVKRSPNMCASVGIQGIAWAFGGMIFALVYCTAGISGGHINPAVTFGLFLARKLSLTRAVYYIVMQCLGAICGAGVVKGFQPKQYQALGGGANTIAHGYTKGSGLGAEIIGTFVLVYTVFSATDAKRNARDSHVPILAPLPIGFAVFLVHLATIPITGTGINPARSLGAAIIFNKDNAWDDHWVFWVGPFIGAALAALYHVIVIRAIPFKSRS*

>AtPIP1;3

MEGKEEDVRVGANKFPERQPIGTSAQTDKDYKEPPPAPFFEPGELSSWSFYRAGIAEFIATFLFLYITVLTVMGVKRAPNMCASVGIQGIAWAFGGMIFALVYCTAGISGGHINPAVTFGLFLARKLSLTRAVFYIVMQCLGAICGAGVVKGFQPNPYQTLGGGANTVAHGYTKGSGLGAEIIGTFVLVYTVFSATDAKRSARDSHVPILAPLPIGFAVFLVHLATIPITGTGINPARSLGAAIIYNKDHAWDDHWIFWVGPFIGAALAALYHQLVIRAIPFKSRS*

>AtPIP1;4

MEGKEEDVRVGANKFPERQPIGTSAQSTDKDYKEPPPAPLFEPGELSSWSFYRAGIAEFIATFLFLYITVLTVMGVKRAPNMCASVGIQGIAWAFGGMIFALVYCTAGISGGHINPAVTFGLFLARKLSLTRAVFYMIMQCLGAICGAGVVKGFQPTPYQTLGGGANTVAHGYTKGSGLGAEIIGTFVLVYTVFSATDAKRSARDSHVPVWTPLLVPILAPLPIGFAVFLVHLATIPITGTGINPARSLGAAIIYNKDHSWDDHWIFWVGPFIGAALAALYHQIVIRAIPFKSKS*

>AtPIP1;5

MEGKEEDVNVGANKFPERQPIGTAAQTESKDYKEPPPAPFFEPGELKSWSFYRAGIAEFIATFLFLYVTVLTVMGVKRAPNMCASVGIQGIAWAFGGMIFALVYCTAGISGGHINPAVTFGLFLARKLSLTRALFYIVMQCLGAICGAGVVKGFQPGLYQTNGGGANVVAHGYTKGSGLGAEIVGTFVLVYTVFSATDAKRSARDSHVPILAPLPIGFAVFLVHLATIPITGTGINPARSLGAAIIYNKDHAWDDHWIFWVGPFIGAALAALYHQIVIRAIPFKSKT*

>AtNIP2;1

MDDISVSKSNHGNVVVLNIKASSLADTSLPSNKHESSSPPLLSVHFLQKLLAELVGTYYLIFAGCAAIAVNAQHNHVVTLVGIAVVWGIVIMVLVYCLGHLSAHFNPAVTLALASSQRFPLNQVPAYITVQVIGSTLASATLRLLFDLNNDVCSKKHDVFLGSSPSGSDLQAFVMEFIITGFLMLVVCAVTTTKRTTEELEGLIIGATVTLNVIFAGEVSGASMNPARSIGPALVWGCYKGIWIYLLAPTLGAVSGALIHKMLPSIQNAEPEFSKTGSSHKRVTDLPL*

>HsAQP1

MASEFKKKLFWRAVVAEFLATTLFVFISIGSALGFKYPVGNNQTTVQDNVKVSLAFGLSIATLAQSVGHISGAHLNPAVTLGLLLSCQISIFRALMYIIAQCVGAIVATAILSGITSSLTGNSLGRNDLADGVNSGQGLGIEIIGTLQLVLCVLATTDRRRRDLGGSAPLAIGLSVALGHLLAIDYTGCGINPARSFGSAVITHNFSNHWIFWVGPFIGGALAVLIYDFILAPRSSDLTDRVKVWTSGQVEEYDLDADDINSRVEMKPK*

>HsAQP2

MWELRSIAFSRAVFAEFLATLLFVFFGLGSALNWPQALPSVLQIAMAFGLGIGTLVQALGHISGAHINPAVTVACLVGCHVSVLRAAFYVAAQLLGAVAGAALLHEITPADIRGDLAVNALSNSTTAGQAVTVELFLTLQLVLCIFASTDERRGENPGTPALSIGFSVALGHLLGIHYTGCSMNPARSLAPAVVTGKFDDHWVFWIGPLVGAILGSLLYNYVLFPPAKSLSERLAVLKGLEPDTDWEEREVRRRQSVELHSPQSLPRGTKA*

>HsAQP3

MGRQKELVSRCGEMLHIRYRLLRQALAECLGTLILVMFGCGSVAQVVLSRGTHGGFLTINLAFGFAVTLGILIAGQVSGAHLNPAVTFAMCFLAREPWIKLPIYTLAQTLGAFLGAGIVFGLYYDAIWHFADNQLFVSGPNGTAGIFATYPSGHLDMINGFFDQFIGTASLIVCVLAIVDPYNNPVPRGLEAFTVGLVVLVIGTSMGFNSGYAVNPARDFGPRLFTALAGWGSAVFTTGQHWWWVPIVSPLLGSIAGVFVYQLMIGCHLEQPPPSNEEENVKLAHVKHKEQI*

>NtPIP1;1

MAENKEEDVNLGANKYRETQPLGTAAQTENKDYIEPPPAPLFEPGELSSWSFYRAGIAEFMATFLFLYITILTVMGLKRSDSLCSSVGIQGVAWAFGGMIFALVYCTAGISGGHINPAVTFGLFLARKLSLTRAVFYMVMQCLGAICGAGVVKGFMKGPYQRLGGGANVVNPGYTKGDGLGAEIIGTFVLVYTVFSATDAKRNARDSHVPILAPLPIGFAVFLVHLATIPITGTGINPARSLGAAIIFNKKQAWDDHWIFWVGPFIGAALAAVYHQIIIRAIPFKS*

>NtPIP1;2

MAENKEEDVKLGANKFRETQPLGTAAQTDKDYKEPPPAPLFEPGELSSWSFYRAGIAEFMATFLFLYITILTVMGLKRSDSLCSSVGIQGVAWAFGGMIFALVYCTAGISGGHINPAVTFGLFLARKLSLTRAIFYIVMQCLGAICGAGVVKGFMVGPYQRLGGGANVVNHGYTKGDGLGAEIIGTFVLVYTVFSATDAKRNARDSYVPILAPLPIGFAVFLVHLATIPITGTGINPARSLGAAIIYNTDQAWDDHWIFWVGPFIGAALAAVYHQIIIRAIPFHKSS*

>NtPIP1;3

MAENKEEDVKLGANKYRETQPLGTAAQTDKDYKEPPPAPLVWRQKSCRHGLFTELEFSMATFLFLYITILTVMGLKRSDSLCSSVGIQGVAWAFGGMIFALVYCTAGISGGHINPAVTFGLFLARKLSLTRAVFYMVMQCLGAICGAGVVKGFMVGPYQRLGGGANVVNPGYTKGDGLGAEIIGTFVLVYTVFSATDAKRNARDSHVPILAPLPIGFAVFLVHLATIPITGTGITPARSLGAAIIFNQDRAWDDHWIFWVGPFIGAALAAVYHQIIIRAIAFKS*

>NtPIP1;4

MAENKEEDVKLGANKFRETQPLGTAAQTDKDYKEPPPAPLFEPGELSSWSFYRAGIAEFMATFLFLYITILTVMGLKRSDSLCSSVGIQGVAWAFGGMIFALVYCTAGISGGHINPAVTFGLFLARKLSLTRAIFYIVMQCLGAICGAGVVKGFMVGPYQRLGGGANVVNHGYTKGDGLGAEIIGTFVLVYTVFSATDAKRNARDSHVPILAPLPIGFAVFLVHLATIPITGTGINPARSLGAAIIYNTDQAWDDHWIFWVGPFIGAALAAVYHQIIIRAIPFHKSS*

>NtPIP2;1

MSKDVIEEGQVHQQHGKDYVDPPPAPLLDFAELKLWSFHRALIAEFIATLLFLYVTVATVIGHKKLNGADKCDGVGILGISWAFGGMIFVLVYCTAGISGGHINPAVTFGLFLARKVSLLRAVGYIIAQSLGAICGVGLVKGFMKHYYNTLGGGANFVQPGYNKGTALGAEIIGTFVLVYTVFSATDPKRSARDSHVPVLAPLPIGFAVFMVHLATIPITGTGINPARTFGAAVIYNTEKIWDDQWIFWVGPFVGALVAAVYHQYILRGSAIKALGSFRSNPTN*

>NtXIP1;1

MASNASHVLGDEESQLSGGSNRVQPFSSTPKNRNIDDEGKKHTSLTVAQRLGISDFFSLDVWRASVGELLGSAVLVFMLDTIVISTFESDVKMPNLIMSILIAIVITILLLAVVPVSGGHINPVISFSAALVGIISMSRAIIYMVAQCVGAILGALALKAVVSSTIAQTFSLGGCTITVIAPGPNGPITVGLEMAQALWLEIFCTFVFLFASIWMAYDHRQAKALGLVTVLSIVGIVLGLLVFISTTVTMKKGYAGAGMNPARCFGAAVVRGGHLWDGHWIFWVGPTIACVAFYVYTKIIPPKHFHADGYKYDFIGVVKASFGLHE*

>LbAQP1

MHPQVASLFDNVYEDLAAATLEFIGTAFFLLFGLGGIQASTAEDTASSQPPASGIEHVLYISTCMGFSLVVSAWLFFRVTGGLFNPNISFALLLVGGLKPLRFVLFCIAQLTGAIAGAAIVRGLTSAPLSVNNVLQQGTSAAQGVFIEMFITAALVLSVLMLAAEKHEATPFAPVGIGLTLFACHLFAVYYTGAAMNSARAFGPAVISGFPEPQHWVYWVGPFLGSLLGAGFYATLKHYKYWRLNPDQATSDYRKSPSDPVALLKSTAETFINVGDEETRNGCASNEEGVRATGDEKSSNATSSRTNFSPV*

>LbAQP3

MSATPIIHLRDVKKRTGVLNAWERVRNKPQVHWAMECFAEALGVFFYVYFGLGSTAAWVIGNILKQSGLSSVFQIGFAYAFGILFAIGVCAATSGGHFNPCVTIAFTIFRGFPPLKAVRYIVAQILGAYIASALVYNQWKVLIVESELLLKQAGVYETTMFTPNGPAGIFALYLLPGAQTLPRAFLNEFVNCFVLALVIWAALDPTSFMIPPVMAPFIIAAAYAGSIWGYAVPAISLNSARDIGCRLFALTIWGKSAAGGSYSAIAALVNIPATLLAAVVYELFLVDSDRVVAGSHLEFMNVAANHRRHRQQAEDDNLVEADDSSQEKPV*

>LbAQP5

MAEFVGVALLVIFGAGAACQVILSTNPGVSPSERGSFLSINFGWAIGIATGAWVSTGMSEGHINPAITIGMATYRGFPWREVPGYIFGQVLGGFVGAALVYANYFHAIDIFEGGHRTQATASLFATFALPYMTQASCFFSEFLATAVLFIVFLALNDKHNGALTNGLLPFALFILFIGLGASLGMQTGYAVNPARDFGPRLFLAMAGYGKAVFNYRRQYWIWAPIIAPILGAQAGGLLYDTFIYNGDDSPIKWR*

>LbAQP6

MDDKFDDDALPNSKTTAKDYEDKLPEYDYTTTFPNTWMRLREPFREYFAEFVGVAVLIIFGVGADCQVVLSANTGVASSPKGSYLSLNCGWAIGTAMGVWISGGISGGHINPAVTLAMATWRGFPWWKVPGFIFAQLLGGIVGAGLVYVNYIHAIDIVEGGRHIRTLDTAGLFATYAADYMTNLSCFFSEFLATAVLIIVIHAMNDKRNTPPPAGIVPFVLFFLILGIGASLGMETGYAINPARDLGPRMLTAMVGYGRQVFAFRNQYWIWCPVLAPFLGAQVGTIFYDLFFYKGQDNVFGRLGSHIHISPA*

>LbAQP7

MSGQHQITEQPSGNPLSRTSTLIQEKPLTPTSSHAETQKHLEAPRQSSFLIQLQDIRHAIRMPMAEFFGVALLIIFGAGSACQVVLSTNPNVASSDRGSFLSINLGWAIGIAMGAWVSGGISGGHINPAITIAMATYRGFPWRRVPSYIFAQVLGGVVGAALVYANYIHAIDIFEGGRHVRTQATASLFATYALPYMTQVSCFFSEFLATAVLSMMVLALTDNRNGAPTNGLLPFALFVLFIGLGASLGMETAYALNPARDFGPRLFLAMSGYGKALFNYRSQYWLWAPIIAPVLGAQAGGLLYDTFLYDGDNSPIKWRRASSQECQLAEVV*

## Note S3 Alignment of amino acid sequences of the aquaporins in this study by CLUSTAL O (V.1.2.1)

LbAQP3 -----------------------------MSATPIIH---------LRDVKKRTGVLNAW HsAQP3 -----------------------------------------MGRQKELVSRCGEM----L LbAQP6 -------MD------DKFDDDAL----------PNSKTTAKDYEDKLPEYDYTTTFPNTW LbAQP5 ------------------------------------------------------------ LbAQP7 -------MSGQHQITEQPSGNPLSRTSTLIQEKPLTPTSSHAETQKHLEAPRQSSFLIQL AtNIP2;1 --------MDDISVSKSNHGNVVVLNI---KASSL-ADTSLPSNKHE----SSS------ NtXIP1;1 MASNASHVLGDEE-SQLSGGSNRVQPF---SST-P-KNRNIDDEGKK----HTSLTVAQR LbAQP1 ----------------------------------------------------MHPQVASL NtPIP2;1 ----------------------MSKDV-----I-E-EGQVHQQHGKD----YVDPPPAPL AtPIP1;5 --------MEGKE-EDVNVGANKFPER-----Q-P-IGTAAQTESKD----YKEPPPAPF AtPIP1;4 --------MEGKE-EDVRVGANKFPER-----Q-P-IGTSAQSTDKD----YKEPPPAPL AtPIP1;3 --------MEGKE-EDVRVGANKFPER-----Q-P-IGTSAQ-TDKD----YKEPPPAPF AtPIP1;1 --------MEGKE-EDVRVGANKFPER-----Q-P-IGTSAQ-SDKD----YKEPPPAPF AtPIP1;2 --------MEGKE-EDVRVGANKFPER-----Q-P-IGTSAQ-SDKD----YKEPPPAPL NtPIP1;3 -------MAENKE-EDVKLGANKYRET-----Q-P-LGTAAQT-DKD----YKEPPPAPL NtPIP1;1 -------MAENKE-EDVNLGANKYRET-----Q-P-LGTAAQTENKD----YIEPPPAPL NtPIP1;2 -------MAENKE-EDVKLGANKFRET-----Q-P-LGTAAQT-DKD----YKEPPPAPL NtPIP1;4 -------MAENKE-EDVKLGANKFRET-----Q-P-LGTAAQT-DKD----YKEPPPAPL HsAQP1 ------------------------------------------------------------ HsAQP2 ------------------------------------------------------------

LbAQP3 ERVRNKPQVHWAMECFAEALGVFFYVYFGLGSTAAWVIGNI--LKQ--SGLSSVFQIGFA HsAQP3 HIRYR-----LLRQALAECLGTLILVMFGCGSVAQVVLSRG--------THGGFLTINLA LbAQP6 MRLRE-----PFREYFAEFVGVAVLIIFGVGADCQVVLSANTGVAS--SPKGSYLSLNCG LbAQP5 ---------------MAEFVGVALLVIFGAGAACQVILSTNPGVSP--SERGSFLSINFG LbAQP7 QDIRH-----AIRMPMAEFFGVALLIIFGAGSACQVVLSTNPNVAS--SDRGSFLSINLG AtNIP2;1 ---PPLLSVHFLQKLLAELVGTYYLIFAGCAAIAVNAQHNH---------VVTLVGIAVV NtXIP1;1 LGISDFFSLDVWRASVGELLGSAVLVFMLDTIVISTFESDV---------KMPNLIMSIL LbAQP1 FDN----VYEDLAAATLEFIGTAFFLLFGLGGIQASTAEDTASSQPPASGIEHVLYISTC NtPIP2;1 LDFAELKLWSFHRALIAEFIATLLFLYVTVATVIGHKKLNG-ADKCD---GVGILGISWA AtPIP1;5 FEPGELKSWSFYRAGIAEFIATFLFLYVTVLTVMGVKRA---PNMCA---SVGIQGIAWA AtPIP1;4 FEPGELSSWSFYRAGIAEFIATFLFLYITVLTVMGVKRA---PNMCA---SVGIQGIAWA AtPIP1;3 FEPGELSSWSFYRAGIAEFIATFLFLYITVLTVMGVKRA---PNMCA---SVGIQGIAWA AtPIP1;1 FEPGELSSWSFWRAGIAEFIATFLFLYITVLTVMGVKRS---PNMCA---SVGIQGIAWA AtPIP1;2 FEPGELASWSFWRAGIAEFIATFLFLYITVLTVMGVKRS---PNMCA---SVGIQGIAWA NtPIP1;3 VWRQKSCRHGLF-TELEFSMATFLFLYITILTVMGLKRS---DSLCS---SVGIQGVAWA NtPIP1;1 FEPGELSSWSFYRAGIAEFMATFLFLYITILTVMGLKRS---DSLCS---SVGIQGVAWA NtPIP1;2 FEPGELSSWSFYRAGIAEFMATFLFLYITILTVMGLKRS---DSLCS---SVGIQGVAWA NtPIP1;4 FEPGELSSWSFYRAGIAEFMATFLFLYITILTVMGLKRS---DSLCS---SVGIQGVAWA HsAQP1 -MASEFKKKLFWRAVVAEFLATTLFVFISIGSALGFKYPVG-NNQTT---VQDNVKVSLA HsAQP2 --MWELRSIAFSRAVFAEFLATLLFVFFGLGSALNWPQA-----------LPSVLQIAMA

.. : :

LbAQP3 YAFGILFAIGVCAATSGGHFNPCVTIAFTIFRGFPPLKAVRYIVAQILGAYIASALVYNQ HsAQP3 FGFAVTLGILIAGQVSGAHLNPAVTFAMCFLAREPWIKLPIYTLAQTLGAFLGAGIVFGL LbAQP6 WAIGTAMGVWISGGISGGHINPAVTLAMATWRGFPWWKVPGFIFAQLLGGIVGAGLVYVN LbAQP5 WAIGIATGAWVSTGMSEGHINPAITIGMATYRGFPWREVPGYIFGQVLGGFVGAALVYAN LbAQP7 WAIGIAMGAWVSGGISGGHINPAITIAMATYRGFPWRRVPSYIFAQVLGGVVGAALVYAN AtNIP2;1 WGIVIMVLVYCLGHLS-AHFNPAVTLALASSQRFPLNQVPAYITVQVIGSTLASATLRLL NtXIP1;1 IAIVITILLLAVVPVSGGHINPVISFSAALVGIISMSRAIIYMVAQCVGAILGALALKAV LbAQP1 MGFSLVVSAWLFFRVTGGLFNPNISFALLLVGGLKPLRFVLFCIAQLTGAIAGAAIVRGL NtPIP2;1 FGGMIFVLVYCTAGISGGHINPAVTFGLFLARKVSLLRAVGYIIAQSLGAICGVGLVKGF AtPIP1;5 FGGMIFALVYCTAGISGGHINPAVTFGLFLARKLSLTRALFYIVMQCLGAICGAGVVKGF AtPIP1;4 FGGMIFALVYCTAGISGGHINPAVTFGLFLARKLSLTRAVFYMIMQCLGAICGAGVVKGF AtPIP1;3 FGGMIFALVYCTAGISGGHINPAVTFGLFLARKLSLTRAVFYIVMQCLGAICGAGVVKGF AtPIP1;1 FGGMIFALVYCTAGISGGHINPAVTFGLFLARKLSLTRALYYIVMQCLGAICGAGVVKGF AtPIP1;2 FGGMIFALVYCTAGISGGHINPAVTFGLFLARKLSLTRAVYYIVMQCLGAICGAGVVKGF NtPIP1;3 FGGMIFALVYCTAGISGGHINPAVTFGLFLARKLSLTRAVFYMVMQCLGAICGAGVVKGF NtPIP1;1 FGGMIFALVYCTAGISGGHINPAVTFGLFLARKLSLTRAVFYMVMQCLGAICGAGVVKGF NtPIP1;2 FGGMIFALVYCTAGISGGHINPAVTFGLFLARKLSLTRAIFYIVMQCLGAICGAGVVKGF NtPIP1;4 FGGMIFALVYCTAGISGGHINPAVTFGLFLARKLSLTRAIFYIVMQCLGAICGAGVVKGF HsAQP1 FGLSIATLAQSVGHISGAHLNPAVTLGLLLSCQISIFRALMYIIAQCVGAIVATAILSGI HsAQP2 FGLGIGTLVQALGHISGAHINPAVTVACLVGCHVSVLRAAFYVAAQLLGAVAGAALLHEI

. : . :** ::.. . : * *. . :

LbAQP3 WKVLIVESELLLKQAGVYETTMFTPNGPAGIFALYLLPGAQTLPRAFLNEFVNCFVLALV HsAQP3 YYDAIWHFAD-------NQLFVSGPNGTAGIFATYPSGHLD-MINGFFDQFIGTASLIVC LbAQP6 YIHAIDIVEG-------GRHI--RTLDTAGLFATYAADYMT-NLSCFFSEFLATAVLIIV LbAQP5 YFHAIDIFEG-------GH----RTQATASLFATFALPYMT-QASCFFSEFLATAVLFIV LbAQP7 YIHAIDIFEG-------GRHV--RTQATASLFATYALPYMT-QVSCFFSEFLATAVLSMM AtNIP2;1 FDLNND---------VCS--------KKHDVFLG-SSPSG-SDLQAFVMEFIITGFLMLV NtXIP1;1 VSSTIAQTFSL---GGCTITV--IAPGPNGP----ITVGL-EMAQALWLEIFCTFVFLFA LbAQP1 TSAPLS----V-----------------NNV----LQQGT-SAAQGVFIEMFITAALVLS NtPIP2;1 MKHYYN----T---LGGG----------ANF----VQPGY-NKGTALGAEIIGTFVLVYT AtPIP1;5 QPGLYQ----T---NGGG----------ANV----VAHGY-TKGSGLGAEIVGTFVLVYT AtPIP1;4 QPTPYQ----T---LGGG----------ANT----VAHGY-TKGSGLGAEIIGTFVLVYT AtPIP1;3 QPNPYQ----T---LGGG----------ANT----VAHGY-TKGSGLGAEIIGTFVLVYT AtPIP1;1 QPKQYQ----A---LGGG----------ANT----VAHGY-TKGSGLGAEIIGTFVLVYT AtPIP1;2 QPKQYQ----A---LGGG----------ANT----IAHGY-TKGSGLGAEIIGTFVLVYT NtPIP1;3 MVGPYQ----R---LGGG----------ANV----VNPGY-TKGDGLGAEIIGTFVLVYT NtPIP1;1 MKGPYQ----R---LGGG----------ANV----VNPGY-TKGDGLGAEIIGTFVLVYT NtPIP1;2 MVGPYQ----R---LGGG----------ANV----VNHGY-TKGDGLGAEIIGTFVLVYT NtPIP1;4 MVGPYQ----R---LGGG----------ANV----VNHGY-TKGDGLGAEIIGTFVLVYT HsAQP1 TSSLTG--------NSLG----------RND----LADGV-NSGQGLGIEIIGTLQLVLC HsAQP2 TPADIR--------GDLA----------VNA----LSNST-TAGQAVTVELFLTLQLVLC

. ::. :

LbAQP3 IWAALDPTSFMIPP-----------VMAPFIIAAAY-AGS--IWGYAVPAISLNSARDIG HsAQP3 VLAIVDPYNNPVPR-----------GLEAFTVGLVV-LVIGTSMGF-NSGYAVNPARDFG LbAQP6 IHAMNDKRNTPPPA-----------GIVPFVLFFLI-LGIGASLGM-ETGYAINPARDLG LbAQP5 FLALNDKHNGALTN-----------GLLPFALFILF-IGLGASLGM-QTGYAVNPARDFG LbAQP7 VLALTDNRNGAPTN-----------GLLPFALFVLF-IGLGASLGM-ETAYALNPARDFG AtNIP2;1 VCAVTTTKRTTE-------------ELEGLIIGATVTLNVI--FAGEVSGASMNPARSIG NtXIP1;1 SIWMAYDHRQAKALGLV-----TVLSIVGIVLGLLVFISTTVTMKKGYAGAGMNPARCFG LbAQP1 VLMLAAEKHEAT-------------PFAPVGIGLTLFACHL--FAVYYTGAAMNSARAFG NtPIP2;1 VFSATDPKRSARDSHVP--------VLAPLPIGFAVFMVHL--ATIPITGTGINPARTFG AtPIP1;5 VFSATDAKRSARDSHVP--------ILAPLPIGFAVFLVHL--ATIPITGTGINPARSLG AtPIP1;4 VFSATDAKRSARDSHVPVWTPLLVPILAPLPIGFAVFLVHL--ATIPITGTGINPARSLG AtPIP1;3 VFSATDAKRSARDSHVP--------ILAPLPIGFAVFLVHL--ATIPITGTGINPARSLG AtPIP1;1 VFSATDAKRNARDSHVP--------ILAPLPIGFAVFLVHL--ATIPITGTGINPARSLG AtPIP1;2 VFSATDAKRNARDSHVP--------ILAPLPIGFAVFLVHL--ATIPITGTGINPARSLG NtPIP1;3 VFSATDAKRNARDSHVP--------ILAPLPIGFAVFLVHL--ATIPITGTGITPARSLG NtPIP1;1 VFSATDAKRNARDSHVP--------ILAPLPIGFAVFLVHL--ATIPITGTGINPARSLG NtPIP1;2 VFSATDAKRNARDSYVP--------ILAPLPIGFAVFLVHL--ATIPITGTGINPARSLG NtPIP1;4 VFSATDAKRNARDSHVP--------ILAPLPIGFAVFLVHL--ATIPITGTGINPARSLG HsAQP1 VLATTDRRRRDLG------------GSAPLAIGLSVALGHL--LAIDYTGCGINPARSFG HsAQP2 IFASTDERRGENP------------GTPALSIGFSVALGHL--LGIHYTGCSMNPARSLA

. : . .:. ** :.

LbAQP3 CRLFA-LTIWGKSAAGGS--YSA-IAALVNIPATLLAAVVYELFLVDSDRVVAGSHL-EF HsAQP3 PRLFTALAGWGSAVFTTG-QHWWWVPIVSPLLGSIAGVFVYQLMIGCHLEQPPPSNE-EE LbAQP6 PRMLTAMVGYGRQVFAFRNQYWIWCPVLAPFLGAQVGTIFYDLFFYKGQDNVFGRLG-SH LbAQP5 PRLFLAMAGYGKAVFNYRRQYWIWAPIIAPILGAQAGGLLYDTFIYNGDDSPIKWR*--- LbAQP7 PRLFLAMSGYGKALFNYRSQYWLWAPIIAPVLGAQAGGLLYDTFLYDGDNSPIKWRR-AS AtNIP2;1 PALVW-------GCY-----KGIWIYLLAPTLGAVSGALIHKMLPSIQNAE--PEFS--K NtXIP1;1 AAVVRGG-----HLW-----DGHWIFWVGPTIACVAFYVYTK-IIPPKHFH-ADGYKYDF LbAQP1 PAVISGF------PE-----PQHWVYWVGPFLGSLLGAGFYATLKH---------YKYWR NtPIP2;1 AAVIYNTE----KIW-----DDQWIFWVGPFVGALVAAVYHQYILRGSAIKALGSFR--- AtPIP1;5 AAIIYNKD----HAW-----DDHWIFWVGPFIGAALAALYHQIVIRAIPFKSKT------ AtPIP1;4 AAIIYNKD----HSW-----DDHWIFWVGPFIGAALAALYHQIVIRAIPFKSKS------ AtPIP1;3 AAIIYNKD----HAW-----DDHWIFWVGPFIGAALAALYHQLVIRAIPFKSRS------ AtPIP1;1 AAIIYNKD----HSW-----DDHWVFWVGPFIGAALAALYHVVVIRAIPFKSRS------ AtPIP1;2 AAIIFNKD----NAW-----DDHWVFWVGPFIGAALAALYHVIVIRAIPFKSRS------ NtPIP1;3 AAIIFNQD----RAW-----DDHWIFWVGPFIGAALAAVYHQIIIRAIAFKS*------- NtPIP1;1 AAIIFNKK----QAW-----DDHWIFWVGPFIGAALAAVYHQIIIRAIPFKS*------- NtPIP1;2 AAIIYNTD----QAW-----DDHWIFWVGPFIGAALAAVYHQIIIRAIPFHKSS*----- NtPIP1;4 AAIIYNTD----QAW-----DDHWIFWVGPFIGAALAAVYHQIIIRAIPFHKSS*----- HsAQP1 SAVIT-------HNF-----SNHWIFWVGPFIGGALAVLIYDFILAPRSSDLTDRVKVWT HsAQP2 PAVVT-------GKF-----DDHWVFWIGPLVGAILGSLLYNYVLFPPAKSLSERLAVLK

:. : . .

LbAQP3 -MNVAANHRRHRQQ---------AEDDNLVEADDSSQE---------------------- HsAQP3 --NVKLAHVKHKEQ---------I*----------------------------------- LbAQP6 IHISPA*----------------------------------------------------- LbAQP5 ------------------------------------------------------------ LbAQP7 SQECQLAEVV*------------------------------------------------- AtNIP2;1 TGS------SHKRVTDLPL----------------------------------------- NtXIP1;1 IGVVKASFGLHE*----------------------------------------------- LbAQP1 LNPDQATSDYRKSP-SDPVALLKSTAETFINVGDEETRNGCASNEEGVRATGDEKSSNAT NtPIP2;1 SNP---TN-*-------------------------------------------------- AtPIP1;5 ------------------------------------------------------------ AtPIP1;4 ------------------------------------------------------------ AtPIP1;3 ------------------------------------------------------------ AtPIP1;1 ------------------------------------------------------------ AtPIP1;2 ------------------------------------------------------------ NtPIP1;3 ------------------------------------------------------------ NtPIP1;1 ------------------------------------------------------------ NtPIP1;2 ------------------------------------------------------------ NtPIP1;4 ------------------------------------------------------------ HsAQP1 SGQVEEYD-L--------------------DADDINSRVEMK--PK*------------- HsAQP2 GLE-PDTD-W--------------------EEREVRRRQSVELHSPQSLPRGTK------

LbAQP3 ------KPV*

HsAQP3 ----------

LbAQP6 ----------

LbAQP5 ----------

LbAQP7 ----------

AtNIP2;1 ----------

NtXIP1;1 ----------

LbAQP1 SSRTNFSPV*

NtPIP2;1 ----------

AtPIP1;5 ----------

AtPIP1;4 ----------

AtPIP1;3 ----------

AtPIP1;1 ----------

AtPIP1;2 ----------

NtPIP1;3 ----------

NtPIP1;1 ----------

NtPIP1;2 ----------

NtPIP1;4 ----------

HsAQP1 ----------

HsAQP2 ---A*

## Note S4 Alignment of amino acid sequences of six O_2_-transporting aquaporins by CLUSTAL O (V.1.2.1)

NPA motifs and Ar/R residues are highlighted in orange and blue, respectively.

NtXIP1;1 MASNASHVLGDEESQLSGGSNRVQPFSSTPKNRNIDDEGKKHTSLTVAQRLGISDFFSLD NtPIP2;1 -----------------MSKDVI------EEGQVHQQHGKDYVDPPPAPLLDFAELKLWS NtPIP1;3 MAE-------NKEEDVKLGANKYRE---TQPLGTAAQTDKDYKEPPPAPLVWRQKSCRHG NtPIP1;4 MAE-------NKEEDVKLGANKFRE---TQPLGTAAQTDKDYKEPPPAPLFEPGELSSWS HsAQP1 ---------------------------------------------------MASEFKKKL HsAQP2 ----------------------------------------------------MWELRSIA

.

NtXIP1;1 VWRASVGELLGSAVLVFMLDTIVISTFES--D--V-KMPNLIMSILIAIVITILLLAVVP NtPIP2;1 FHRALIAEFIATLLFLYVTVATVIGHKKLNGADKCDGVGILGISWAFGGMIFVLVYCTAG NtPIP1;3 LF-TELEFSMATFLFLYITILTVMGLKRS--DSLCSSVGIQGVAWAFGGMIFALVYCTAG NtPIP1;4 FYRAGIAEFMATFLFLYITILTVMGLKRS--DSLCSSVGIQGVAWAFGGMIFALVYCTAG HsAQP1 FWRAVVAEFLATTLFVFISIGSALGFKYPVGNNQTTVQDNVKVSLAFGLSIATLAQSVGH HsAQP2 FSRAVFAEFLATLLFVFFGLGSALNWPQA-------LPSVLQIAMAFGLGIGTLVQALGH

. : . :.: ::::. .: :: :. * * .

NtXIP1;1 VSGGHINPVISFSAALVGIISMSRAIIYMVAQCVGAILGALALKAVVSSTIAQTFSLGGC NtPIP2;1 ISGGHINPAVTFGLFLARKVSLLRAVGYIIAQSLGAICGVGLVKGFMKHYYN----TLGG NtPIP1;3 ISGGHINPAVTFGLFLARKLSLTRAVFYMVMQCLGAICGAGVVKGFMVGPYQ----RLGG NtPIP1;4 ISGGHINPAVTFGLFLARKLSLTRAIFYIVMQCLGAICGAGVVKGFMVGPYQ----RLGG HsAQP1 ISGAHLNPAVTLGLLLSCQISIFRALMYIIAQCVGAIVATAILSGITSSLT-----GNSL HsAQP2 ISGAHINPAVTVACLVGCHVSVLRAAFYVAAQLLGAVAGAALLHEITPADI-----RGDL

:**.*:**.::.. : :*: ** *: * :**: .. : .

NtXIP1;1 TITVIAPGPNGPITVGLEMAQALWLEIFCTFVFLFASIWMAYDHRQAKALGLVTVLSIVG NtPIP2;1 GANFVQP--------GYNKGTALGAEIIGTFVLVYTVFS-ATDPKRSARDSHVPVL--AP NtPIP1;3 GANVVNP--------GYTKGDGLGAEIIGTFVLVYTVFS-ATDAKRNARDSHVPIL--AP NtPIP1;4 GANVVNH--------GYTKGDGLGAEIIGTFVLVYTVFS-ATDAKRNARDSHVPIL--AP HsAQP1 GRNDLAD--------GVNSGQGLGIEIIGTLQLVLCVLA-TTDRRRRD----LGGS--AP HsAQP2 AVNALSN--------STTAGQAVTVELFLTLQLVLCIFA-STDERRGE----NPGT--PA

. : . . .: *:: *: :: : : * ::

NtXIP1;1 IVLGLLVFISTTVTMKKGYAGAGMNPARCFGAAVVR-GGHLWDGHWIFWVGPTIACVAFY NtPIP2;1 LPIGFAVFMVHL--ATIPITGTGINPARTFGAAVIYNTEKIWDDQWIFWVGPFVGALVAA NtPIP1;3 LPIGFAVFLVHL--ATIPITGTGI**TPA**RSLGAAIIFNQDRAWDDHWIFWVGPFIGAALAA NtPIP1;4 LPIGFAVFLVHL--ATIPITGTGINPARSLGAAIIYNTDQAWDDHWIFWVGPFIGAALAA HsAQP1 LAIGLSVALGHL--LAIDYTGCGINPARSFGSAVIT---HNFSNHWIFWVGPFIGGALAV HsAQP2 LSIGFSVALGHL--LGIHYTGCSMNPARSLAPAVVT---GKFDDHWVFWIGPLVGAILGS

: :*: * : :* .:.*** :. *:: :. :*:**:** :.

NtXIP1;1 VYTK-IIPPKHFHADGYKYDFIGVVKASFGLHE*----------------------- NtPIP2;1 VYHQYILRGSAIKALGSFRSN---P---------------------TN*-------- NtPIP1;3 VYHQIIIRAIAFKS*------------------------------------------ NtPIP1;4 VYHQIIIRAIPFHKSS*----------------------------------------

HsAQP1 LIYDFILAPRSSDLTDRVKVWTSGQVEEYDLDADDINSRVEMK--PK*---------

HsAQP2 LLYNYVLFPPAKSLSERLAVLKGLE-PDTDWEEREVRRRQSVELHSPQSLPRGTKA*

: . ::
